# STAT3/5 Inhibitors Suppress Proliferation in Bladder Cancer and Enhance Oncolytic Adenovirus Therapy

**DOI:** 10.3390/ijms21031106

**Published:** 2020-02-07

**Authors:** Sruthi V. Hindupur, Sebastian C. Schmid, Jana Annika Koch, Ahmed Youssef, Eva-Maria Baur, Dongbiao Wang, Thomas Horn, Julia Slotta-Huspenina, Juergen E. Gschwend, Per Sonne Holm, Roman Nawroth

**Affiliations:** 1 Department of Urology, Klinikum rechts der Isar, Technical University of Munich, 81675 Munich, Germany; sruthi.hindupur@tum.de (S.V.H.); sebastian.schmid@tum.de (S.C.S.); jana.koch@tum.de (J.A.K.); ahmed0122@med.bsu.edu.eg (A.Y.); e-m.baur@gmx.de (E.-M.B.); dongbiao.wang@tum.de (D.W.); t.horn@tum.de (T.H.); juergen.gschwend@tum.de (J.E.G.); per.holm@tum.de (P.S.H.); 2 Department of Urology, Beni-suef University, Beni Suef 62511, Egypt; 3 Department of Pathology, Klinikum rechts der Isar, Technical University of Munich, 81675 Munich, Germany; julia.slotta-huspenina@tum.de

**Keywords:** bladder cancer, JAK-STAT pathway, combination therapy, oncolytic adenovirus, virotherapy, STAT3/5 inhibitor, JAK inhibitor, XVir-N-31

## Abstract

The JAK-STAT signalling pathway regulates cellular processes like cell division, cell death and immune regulation. Dysregulation has been identified in solid tumours and STAT3 activation is a marker for poor outcome. The aim of this study was to explore potential therapeutic strategies by targeting this pathway in bladder cancer (BC). High STAT3 expression was detected in 51.3% from 149 patient specimens with invasive bladder cancer by immunohistochemistry. Protein expression of JAK, STAT and downstream targets were confirmed in 10 cell lines. Effects of the JAK inhibitors Ruxolitinib and BSK-805, and STAT3/5 inhibitors Stattic, Nifuroxazide and SH-4-54 were analysed by cell viability assays, immunoblotting, apoptosis and cell cycle progression. Treatment with STAT3/5 but not JAK1/2 inhibitors reduced survival, levels of phosphorylated STAT3 and Cyclin-D1 and increased apoptosis. Tumour xenografts, using the chicken chorioallantoic membrane (CAM) model responded to Stattic monotherapy. Combination of Stattic with Cisplatin, Docetaxel, Gemcitabine, Paclitaxel and CDK4/6 inhibitors showed additive effects. The combination of Stattic with the oncolytic adenovirus XVir-N-31 increased viral replication and cell lysis. Our results provide evidence that inhibitors against STAT3/5 are promising as novel mono- and combination therapy in bladder cancer.

## 1. Introduction

Bladder cancer (BC) is the 10th most common cancer in the world, as of 2018 [1]. Approximately 25% of patients present as muscle invasive bladder cancer (MIBC) at time of diagnosis. Metastasized BC patients face a poor outcome with median survival time of approximately 14 months and the survival rates have remained largely unchanged for the past 30 years until the emergence of immune checkpoint inhibitors (ICB). Checkpoint inhibitors targeting programmed cell death protein 1 (PD-1) or programmed cell death 1 ligand 1 (PD-L1) have demonstrated durable responses in patients with cisplatin-refractory metastasized BC [2]. However, only 13–21% of patients with metastatic BC respond to immune checkpoint inhibition [3,4,5]. 

To find new therapeutic approaches, a plethora of compounds are being tested in clinical trials targeting various signalling pathways in MIBC, including inhibitors of EGFR, HER2, VEGF and the PI3K/AKT/mTOR pathway [6]. Clinical trials so far have demonstrated that only sub-cohorts of patients benefit from those treatment strategies, probably due to required molecular alterations for therapy response [6]. In 2019, the FGFR inhibitor Balversa (Erdafitinib) was approved by the FDA (Food and Drug Administration) for treatment of patients with metastatic bladder cancer harbouring molecular alterations in FGFR2/3, marking it to be the first targeted therapy to be approved in bladder cancer [7]. Most targeted therapies have shown inconsistent results in early phase clinical trials, which could be attributed to suboptimal selection criteria and biomarker selection, and molecular heterogeneity of the disease [6,8]. Thus, it is not only necessary to identify suitable molecular targets but also predictive markers and combination therapies that broaden the spectrum of responders to treatment. 

The JAK-STAT pathway is one of the most studied pathways in cellular signal transduction with diverse roles in physiological processes including cell growth and differentiation and immune response via cytokine signalling [9]. Dysregulations in this pathway are implicated in carcinogenesis and are also associated with poor prognosis in various cancers including kidney cancer [10], lung cancer [11], cervical cancer [12] and bladder cancer [13,14]. The JAK (Janus Kinase) protein family consists of JAK1-3 and Tyk2 in mammals. These proteins are non-receptor tyrosine kinases that are associated with transmembrane cytokine receptors. Signalling is initiated upon binding of a wide range of cytokines (interleukins, interferons, growth factors) to their appropriate receptor. This leads to the dimerization of receptors, bringing the JAKs into close proximity and facilitating transphosphorylation. These activated JAK proteins mediate phosphorylation of STAT proteins. The STAT (signal transducer and activator of transcription) protein family comprises of 7 family members (STAT-1, 2, 3, 4, 5a, 5b and 6). The phosphorylated state results in dimerization and eventual translocation to the nucleus where STATs can activate transcription of target genes. STAT3, in particular, plays a role in cell cycle regulation, cytokine signalling and apoptosis. Increased protein level and constitutive activation of STAT3 have also been reported in bladder cancer [13,14,15,16,17]. Elevated and increased nuclear level of STAT3 and p-STAT3 have been reported to be associated with invasiveness of the disease and advanced stages of the cancer [13,17,18,19,20,21]. Using a STAT3 transgenic mouse model, chemical induction of bladder cancer by N-butyl-N-(4-Hydroxybutyl) nitrosamine (BBN) directly resulted in the development of invasive carcinoma from carcinoma in situ, asserting the role of STAT3 in bladder cancer progression [18].

Considering these findings, we explored targeting of the JAK1/2 and STAT3/5 proteins using specific small molecule inhibitors and combined these inhibitors with standard chemotherapy, inhibitors of cell cycle progression and oncolytic adenovirus in preclinical models of bladder cancer. Several inhibitors for JAK and STAT proteins have been developed and JAK inhibitors were approved by the FDA for treatment of auto-immune disorders and myelofibrosis [22,23]. STAT3 inhibitors have shown anti-tumour activity in pre-clinical stages and have been successfully tested in phase-I clinical trials for safety and efficacy in solid tumours [24,25].

Application of targeted therapies as monotherapy has been shown to be largely influenced by a specific genetic background of patients or acquired resistance mechanisms in the clinical setting. These limitations can be improved by the use of combination therapies. STAT3 is implicated in the development of chemotherapy resistance in various cancers. Targeting STAT3 in combination with chemotherapeutics sensitises cells to chemotherapy in oral squamous cell carcinoma [26]. The interference with STAT3 activity has also been linked to cell cycle arrest either in G0/1 or in G2 phase [27,28]. Thus, we addressed the question if the combination of STAT3/5 inhibitors and inhibitors of cell cycle progression might be beneficial for therapy response. Targeted therapies against CDK4/6 pathway in bladder cancer showed promising data in various preclinical studies [29]. CDK4/6 inhibitors were approved by the FDA for the treatment of Hormone Receptor (HR)-positive and Human Epidermal Growth Factor Receptor 2 (HER2)-negative breast cancer. However, most patients inevitably develop resistance for multifactorial reasons [30,31]. Recently, our group demonstrated that the JAK-STAT pathway plays a role in acquired resistance of CDK4/6 inhibitors in bladder cancer [32]. In this study, we extended these data to show the efficiency of CDK4/6 inhibition by combining it with STAT3/5 inhibitors.

In recent years, there has been a surge in oncolytic virotherapy with several oncolytic viruses showing anti-cancer activity in patients [33]. The use of oncolytic viruses is a very promising new strategy in which native or modified viruses selectively target cancer cells and cause tumour elimination not only by viral spread but also by elicitation of an anti-tumour immune response [34]. As one example, Talimogene laherparepvec (T-Vec), a modified type I Herpes simplex virus for the treatment of advanced melanoma, was approved by the FDA [35]. A multitude of viruses from vesicular stomatitis virus, vaccinia virus or adenovirus are at various stages in clinical trials and most of them have a remarkable safety profile. Most side effects are infection-related or nausea and are easily manageable [36]. At preclinical level, it has been shown that oncolytic adenoviruses are successful in treating MIBC [37]. Several other oncolytic viruses have shown efficacy in urothelial carcinomas and some are currently tested in clinical trials [38]. Combination of oncolytic viruses with immune checkpoint inhibitors and targeted therapies has been proven to be a successful strategy to enhance the efficiency of therapy response to oncolytic virus [39,40]. There are studies showing that targeting the JAK-STAT pathway in combination with the oncolytic herpes simplex virus and vesicular stomatitis viruses could enhance their efficiency possibly by modulating IFN signalling [41,42]. Hence, in this study we investigated also the effects upon combining oncolytic adenovirus with STAT3/5 inhibitors in bladder cancer.

## 2. Results

### 2.1. JAK-STAT Pathway is Dysregulated in Bladder Cancer

**Figure 1:**
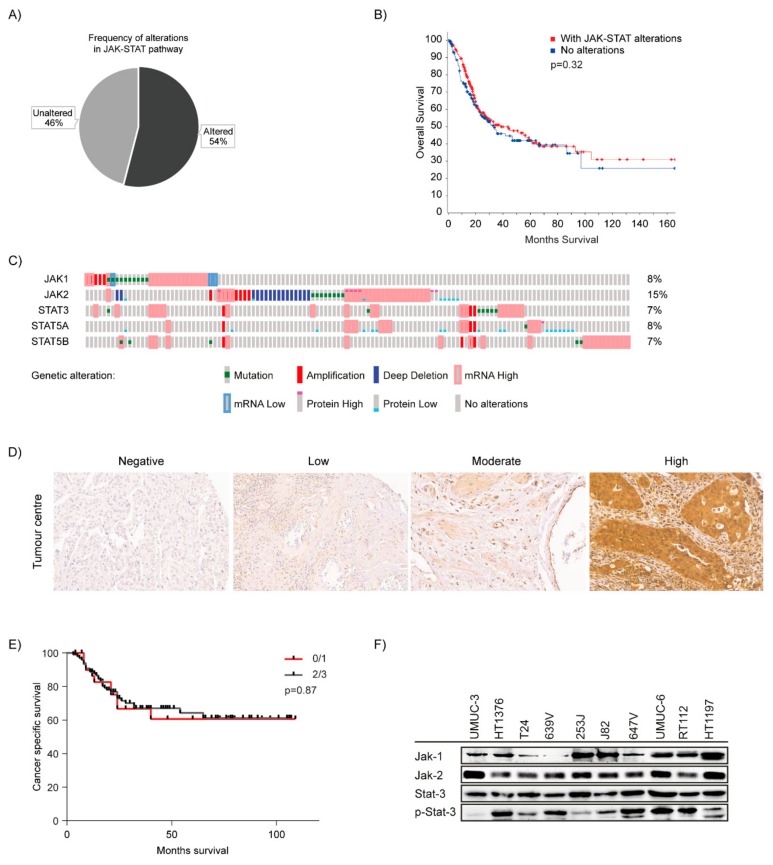
JAK-STAT pathway in bladder cancer: (**A**) TCGA cohort of 412 patients (413 samples) was analysed using cBioPortal. JAK-STAT pathway was altered in 54% of bladder cancer specimens. (**B**) Kalpan-Meier plot depicting overall survival analysis among patients with and without alterations in JAK-STAT pathway in the TCGA cohort. (**C**) Alterations in JAK1, JAK2, STAT3, STAT5A and STAT5B genes—OncoPrint indicates tumours altered with mutations (green bars), amplification (red bars), homozygous deletion (blue bars), high mRNA (red—outlined bars), mRNA low (blue-outlined bars), protein high (bars with red cap), protein low (blue-bottomed bars) and no alterations (grey bars). (**D**) Immunohistochemistry of patient tissues stained for STAT3- images showing staining intensities- Negative (Score-0), Low (Score-1), Moderate (Score-2), High (Score-3). Tissue sections were imaged at 200× magnification. (**E**) Kaplan-Meier plots for cancer specific survival analysis among patients with no or low STAT3 staining vs. moderate/high STAT3 staining. (**F**) Cell lines were analysed for Jak1, Jak2, Stat3 expression and Stat3 phosphorylation by immunoblotting.

Activation of JAK-STAT signalling, mostly by phosphorylation and protein level of STAT3/5 has been described in bladder cancer by several groups [14,15,20,43,44]. We wanted to specifically focus on involvement of JAK-STAT proteins as prognostic marker and also as potential targets for therapy. Thus, we included the 4 JAK family proteins and 7 STAT family proteins and analysed the data in the TCGA cohort consisting of 412 bladder cancer patients to identify molecular alterations in the JAK-STAT signalling pathway related genes (Table S1) [45]. In 54% of the patients, we found alterations in the selected genes (Figure 1A). The alterations in the JAK-STAT pathway did not have a significant effect on overall survival in patients with bladder cancer (Figure 1B). The TCGA cohort analysis showed that alterations in STAT3 were observed in 7%, STAT5A in 8% and STAT5B in 7% of patients. Alterations in JAK1 were observed in 8% and in JAK2 in 15% of patients indicating, that these genes are frequently altered in bladder cancer (Figure 1C). STAT3 and STAT5 are shown to interact majorly with JAK1 and 2. Statistically significant co-occurrence was found in between JAK2-STAT5A, STAT3-STAT5A and STAT3-STAT5B among others (Table S2). As for mutual exclusivity, none of the genes showed any significance.

Subsequently, considering the TCGA analysis results, we also performed immunohistochemistry to determine STAT3 expression. Here, we wanted to confirm that increased expression level of STAT3 protein might serve as a prognostic marker. We used a previously described tissue microarray including tissue specimen from urothelial carcinoma patients with metastatic disease limited to lymph nodes from 149 patients from January 2003 until December 2012. The clinical characteristics of the patients are described in a prior publication [46]. We analysed STAT3 staining in sections from tumour centre. In total, 14/149 showed no staining, 18/149 showed low intensity staining, 34/149 had moderate staining and 74/149 had high intensity staining (Figure 1D). It was established that 9/149 sections were not evaluable due to a technical reason. When correlating the expression with cancer specific survival, we could not observe any significant difference in survival among no/low staining vs. moderate/high staining (Figure 1E). This also correlates with the survival data from the TCGA cohort. However, the high number of patients positively expressing STAT3 qualifies this protein as a potential molecular target.

Inhibitors against JAK1/2 and STAT3/5 are most advanced in clinical use and molecular alterations in STAT3 and STAT5 genes have previously been shown to be altered in various solid cancers [10,12,47,48]. Based on the TCGA results, we decided to analyse expression and activation level of JAK1/2 and STAT3 in a panel of 10 different bladder cancer derived cell lines. Constitutive expression of JAK1/2 protein was detected in all cell lines (Figure 1G). However, phosphorylation of JAK1/2 could not be detected. In all cell lines, expression of STAT3 protein and also its constitutive phosphorylation was detected although at different level in between cell lines.

### 2.2. JAK1/2 Inhibitors Have No Effect on Proliferation in Bladder Cancer Cell Lines

**Figure 2:**
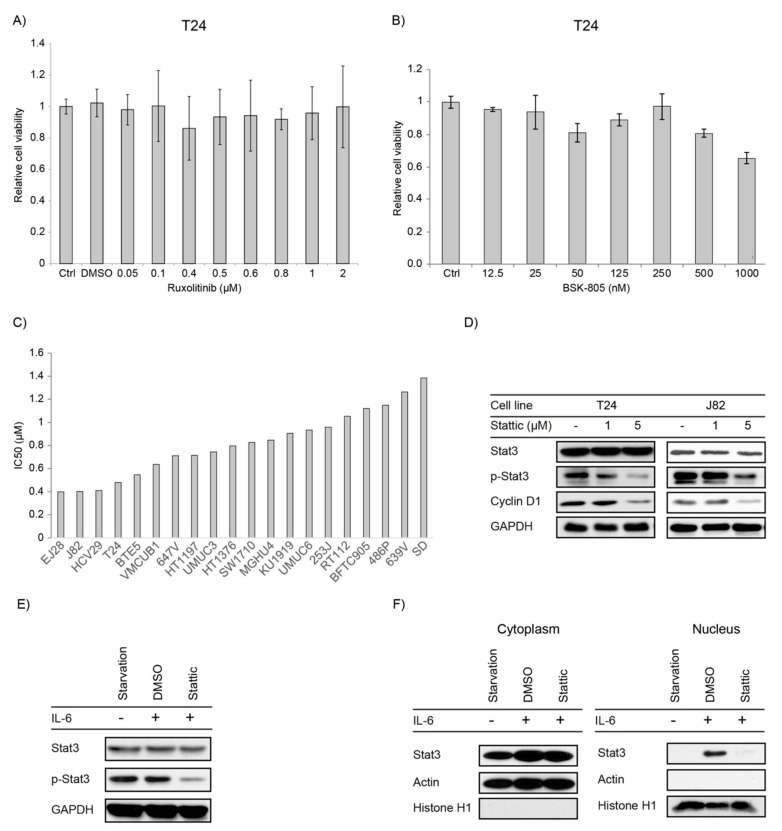
**A**) JAK inhibition by specific inhibitors: T24 cells were treated with increasing concentrations of (**A**) Ruxolitinib and (**B**) BSK-805 and cell viability was assessed by CellTiter-Blue^®^ Cell Viability Assay 72 h after treatment. Error bars S.E. STAT3 inhibition by Stattic: (**C**) Cell viability was assessed by CellTiter-Blue^®^ Cell Viability Assay 72 h after treatment with Stattic and IC50 was determined. Cell lines arranged according to IC50 values. (**D**) T24 and J82 cells were starved for 4 h then activated with 25% FBS and simultaneously treated with serial concentrations of Stattic and DMSO as a control followed by immunoblotting. (**E**) T24 cells were serum starved overnight, treated with 5 µM Stattic (or DMSO) for 3 hours then activated with IL-6 (25 ng/mL) for 30 min before being harvested; protein expression and phosphorylation were analysed by Immunoblotting. (**F**) Cells were treated the same as in (**E**). Compartmental protein extraction was performed, and the lysates were analysed by Immunoblotting.

We wanted to explore whether upstream inhibition of the JAK-STAT pathway would affect cell viability and used the inhibitors Ruxolitinib targeting JAK1/2 and BSK805 which is a specific JAK2 inhibitor. The effect of these drugs on cell viability was tested in a bladder cancer cell panel of 10 different cell lines. Cells were treated with serial concentrations of Ruxolitinib (0.015–10 µM) and BSK805 (0.0125 to 1 µM). Ruxolitinib did not have any effect on cell viability (Figure 2A and Figure S1). The JAK2 inhibitor, BSK-805 diminished cell growth only on the cell lines T24, J82 and RT112, whereas the other cell lines did not respond (Figure 2B and Figure S2). Overall, the inhibition of JAK1/2 proteins did not substantially interfere with cell growth which might be due to the observation that JAK1/2 protein was also not phosphorylated and thus not activated.

### 2.3. STAT3/5 Inhibitors Reduced Proliferation and Downstream Signaling in Bladder Cancer Cell Lines

We also examined effects of STAT3/5 inhibitors on bladder cancer cell growth. Therefore, we applied the specific STAT3/5 inhibitors Stattic and SH-4-54 to 10 different bladder cancer cell lines, and Nifuroxazide in T24 and RT112 cell lines. All inhibitors resulted in dose dependent reduction in cell proliferation in the cell lines tested (Figures S3 and S4). As for Stattic, we extended the number of cell lines to 20 and all cell lines showed a dose-dependent response to Stattic treatment (Figure 2C and Figure S5).

We investigated also the molecular response to Stattic treatment by examining protein levels of total and phosphorylated STAT3. Therefore, we first examined the effect of STAT3 on the constitutive phosphorylation level on T24 and J82 cells which could be suppressed upon treatment in a dose dependent manner (Figure 2D). This effect of Stattic was also confirmed by analysis of the STAT3 downstream effector molecule Cyclin D1, which was downregulated upon treatment.

We also confirmed activity of Stattic on phosphorylation level of STAT3 and its subcellular localization upon an extracellular stimulus, using IL-6. Cells were serum starved, followed by interleukin-6 (IL-6) stimulation. IL-6 stimulation did not result in a further increase in the constitutive STAT3 phosphorylation, but STAT3 phosphorylation could still be suppressed by Stattic (Figure 2E). We then tested the nuclear translocation of STAT3 upon Stattic treatment to evaluate the molecular mechanism of Stattic inhibition. Nuclear and cytoplasmic protein fractions were isolated and the level of total STAT3 and activated STAT3 were analysed. As for control of the purity of the protein compartments, presence of Histone H1 and actin was examined. Interestingly, only after IL-6 treatment we detected a nuclear translocation of STAT3 that was suppressed by Stattic (Figure 2F).

### 2.4. Stattic Induced G2/M Arrest and Apoptosis in Bladder Cancer Cell Lines

**Figure 3:**
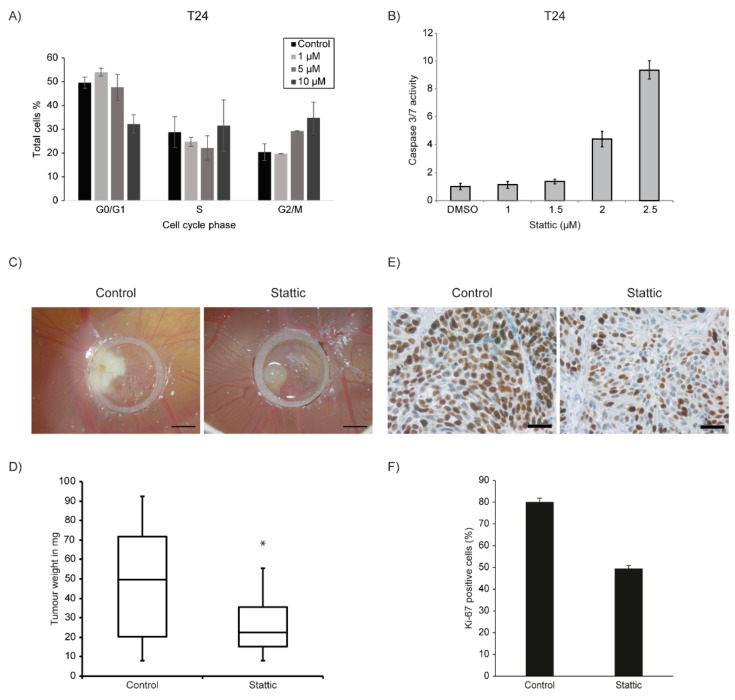
(**A**) Analysis of cell cycle progression in T24 treated with the indicated concentrations of Stattic for 24 h. An increase in G2 population was found with increasing concentration. (**B**) Caspase 3/7 assay for the apoptosis in T24 cells incubated for 24 h with the indicated concentrations of Stattic. The percentage of apoptotic cells was increased in a dose-dependent manner. (**C**) Stattic reduces the bladder cancer cell growth in vivo. Representative images of RT112 cells show tumour formation of Stattic versus control group on the CAM inside a silicone ring, 6 days after seeding of cells. Scale bar equals 2 mm. (**D**) Tumours from indicated cell line were harvested and weighed after Stattic (*n* = 21) or control (DMSO) (*n* = 19) treatment (‘*’ indicates *p* = 0.0069). (**E**) Tissue sections from the tumours were stained for Ki-67. (**F**) Number of Ki-67 positive cells were counted and compared between Control (DMSO) and Stattic-treated groups. Scale bar equals 4 µm. Error bars S.E.

STAT3 and 5 have diverse roles in cells including cell cycle regulation and apoptosis [49]. To investigate whether Stattic influences cell cycle in bladder cancer cells, we treated T24 with Stattic in increasing doses and performed cell cycle analysis measuring BrdU incorporation by flow cytometry, 24 h after treatment. We observed that Stattic induced a G2/M cell cycle arrest in cells (Figure 3A). We also investigated whether Stattic induces apoptosis in a concentration kinetic using T24 cells. Thus, 24 h after treatment, a dose dependent 9-fold increase in Caspase3/7 activity was determined (Figure 3B). From the above results, it is implied that Stattic is acting as a cytostatic and a cytotoxic agent in bladder cancer.

### 2.5. Stattic Reduced Tumour Growth in 3-Dimensional Xenografts

To further test the effect of Stattic on tumour xenografts, we used the chicken chorioallantoic membrane (CAM) model. As for the purpose of this study, this model reflects characteristics of an immunocompromised mouse model, including the development of a host derived vasculature and extracellular matrix [50]. Hence, we tested the effect of Stattic in vivo by using the CAM model to grow three-dimensional in vivo xenografts of RT112 cells. T24 cells could not be used in the CAM assay as they do not grow well and form very small tumours on the CAM. A significant weight reduction in tumour xenografts, reflecting tumour growth reduction of up to 50% was observed upon treatment with Stattic (Figure 3C). We also performed Ki-67 staining on the CAM tumour tissue sections to estimate the Stattic effect on tumour proliferation in the xenografts. A significant decrease in Ki-67 positive cells was observed in the Stattic treated tumours as compared to untreated tumours (Figure 3D). This correlates with the observed decrease in tumour cell proliferation in vitro.

### 2.6. Combination of Stattic and Chemotherapeutic Agents Showed Additivity

STAT3 has been shown to be activated in response to chemotherapeutic agents and mediating drug-resistance in several cancers. Hence, we wanted to explore potential options for combination therapy with Stattic and standard chemotherapeutic drugs. We chose 5 bladder cancer cell lines (HT1376, J82, RT112, SD and T24) with different sensitivities to Stattic monotherapy: J82 as one of the best responder cell lines to Stattic, T24, HT1376 and RT112 as intermediate responder cell lines and SD as lowest responding cell line.

**Figure 4:**
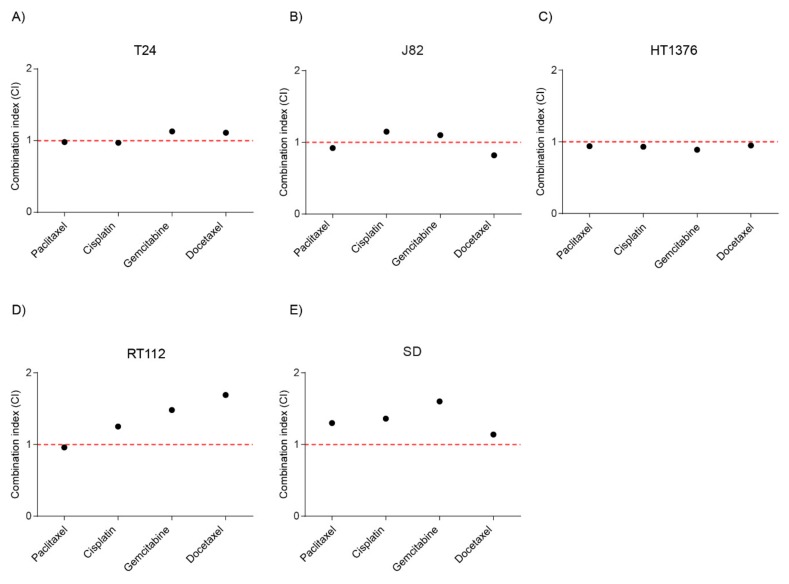
Combination therapy with Stattic and chemotherapeutics. Treatment of the respective cell lines was done for 72 h with Stattic alone and in a fixed ratio combination with Paclitaxel, Cisplatin, Gemcitabine or Docetaxel in (**A**) T24, (**B**) J82, (**C**) HT1376, (**D**) RT112, and (**E**) SD cell lines (See Materials and methods). Combination index (CI) for combining Stattic with Paclitaxel, Cisplatin, Gemcitabine or Docetaxel were plotted. Values were calculated using the Chou-Talalay theorem. CI < 1: synergy, CI = 1; additivity, CI > 1: antagonism.

The cell viability of bladder cancer cell lines in response to the increasing doses of single drugs and combination treatment of Stattic and Cisplatin, Docetaxel, Gemcitabine or Paclitaxel was investigated using the CellTiter-Blue^®^ Cell Viability Assay. Dose response curves for the mono- and combination therapy were generated (Figure S6). Data were analysed using the Chou-Talalay theorem to generate a combination index (CI). According to the theorem, CI values less than 1 indicate a synergistic interaction, while values equal to or greater than 1 indicate additive or antagonistic effects respectively [51]. The combination index was calculated for the combination causing 50% inhibition of cell viability (Fa50). In HT1376 cells, the combination index for all the combinations of Stattic and chemotherapeutic agents were in the range of 1 indicating additivity. In SD cells, the combination index for most of the combinations of Stattic and chemotherapeutic agents were more than 1 indicating antagonism. In T24, J82 and RT112 cells, the combination index varies from additivity to antagonism depending on the combinations (Figure 4).

### 2.7. Combination of Stattic and CDK4/6 inhibitor Palbociclib Showed Additivity

**Figure 5:**
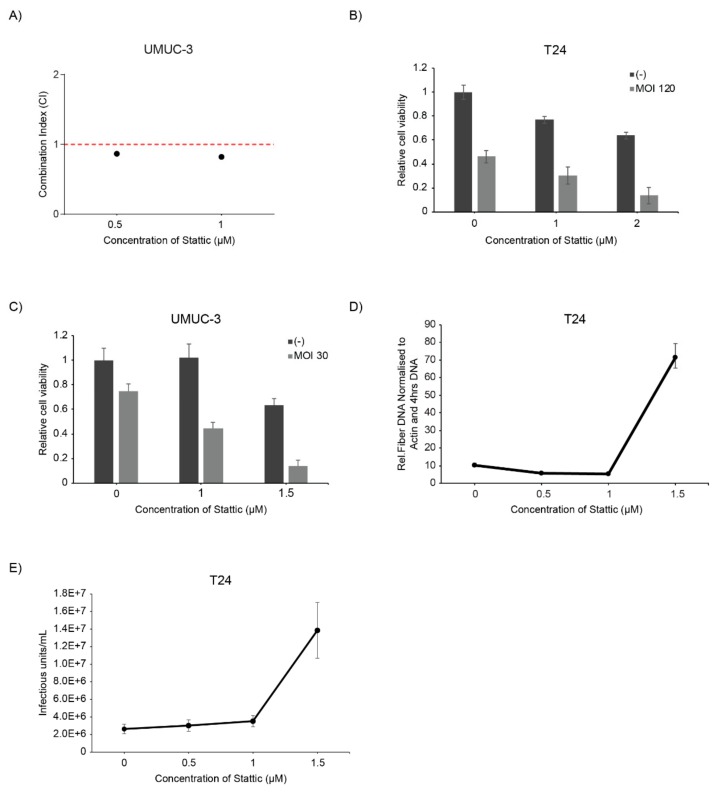
Combination therapy with Stattic and Palbociclib, and oncolytic virotherapy. (**A**) Treatment of the UMUC-3 cells was done for 72 h with Stattic alone at 0.5 µM and 1 µM and in a fixed concentration combination with 0.5 µM Palbociclib. Combination index (CI) for combining Stattic with Palbociclib were plotted. Values were calculated using the Chou-Talalay theorem. (**C**). Cells were treated with Stattic and infected with XVir-N-31 with corresponding multiplicity of infections (MOI) in T24 (**B**) and in UMUC-3 cells (**C**). The MOIs are indicated in the graphs. Cell viability was analysed at 4dpi by sulphorhodamine B assay. Error bars S.E. (**D**,**E**) T24 cells were infected with XVir-N-31 (MOI 50) and treated with increasing concentrations of Stattic (**D**) Viral replication was analysed at 48 h by qPCR of fibre DNA, and (**E**) the increase in viral titre upon Stattic inhibition is measured by hexon titre test and represented as infectious units/mL (IFU/mL).

Our group has previously shown that STAT3 is one of the proteins contributing to therapy resistance to the CDK4/6 inhibitor Palbociclib. Palbociclib induces a G1 arrest in cells. The rational to combine both inhibitors was to induce both G1 and G2 arrest and using Stattic in a supportive role for enhancing Palbociclib activity. We tested this combination before in RT112 and T24 bladder cancer cell lines with slightly controversial results and included here additionally UMUC-3 cells [32]. UMUC-3 cells were treated with increasing doses of Stattic and a fixed concentration of Palbociclib and cell viability was assessed by sulphorhodamine-B assay and the CI was calculated. The combination index was around 1 indicating additive effect of the combination therapy (Figure 5A). In conclusion, the combination of both compounds results in additive activity but requires obviously also specific genetic predisposition as for substantial response limiting it to a subset of patients.

### 2.8. Stattic Enhanced Oncolytic Virotherapy of the Oncolytic Adenovirus XVir-N-31

Oncolytic virotherapy only recently entered clinical application and received FDA approval with a very favourable response rate and low adverse events [37]. It has been shown that the JAK-STAT pathway is activated upon adenoviral infection. As for viruses, such as vesicular stomatitis virus (VSV), inhibition of JAK by Ruxolitinib has been shown to support viral replication [41]. We combined Stattic with oncolytic adenovirus XVir-N-31 to suppress activation of STAT3/5 and analysed the effect on cell viability [37]. Cells were infected with XVir-N-31 24 h after treatment with Stattic. The combination treatment resulted in enhanced virus-induced cell death in both T24 and UMUC-3 cell lines (Figure 5B). We also observed increased viral replication in combination with Stattic which corresponded with an increase in viral particle formation (Figure 5C,D). Thus, inhibition of STAT3/5 substantially supports replication and viral particle production in cancer cells.

## 3. Discussion

In this study, we analysed the potential use of targeting the JAK-STAT pathway for therapy in bladder cancer. It has been observed that expression level of STAT3 correlates with invasion and poor prognosis in bladder cancer [17,18,19,44]. However, other groups could not confirm the prognostic value of STAT3 expression level [14]. We analysed molecular alterations in JAK/STAT proteins in the TCGA dataset including 412 patient specimens of muscle invasive bladder cancer. This analysis revealed that genetic alterations in the molecular members of the JAK-STAT pathway are frequently found in bladder cancer but they are not suitable markers for prognosis. Analysis of STAT3 protein expression level on a TMA confirmed that most primary tumours of muscle invasive disease express high level of STAT3 but in this cohort STAT3 failed to be a prognostic marker for survival. In conclusion, the data presented here question the prognostic value of STAT3 in bladder cancer.

Previously, the JAK2 tyrosine kinase inhibitor AG490 has been tested in bladder cancer with positive results [52]. We wanted to further analyse if these data could be confirmed using the JAK1/2 inhibitor Ruxolitinib and the specific JAK2 inhibitor BSK-805. Surprisingly, our data are in contradiction to the described observations as we do not observe anti-tumour effects of JAK1/2 inhibition on cell proliferation. An explanation for the observed difference is the specificity of the inhibitors used. AG490 is a tyrosine kinase inhibitor that targets molecules such as EGFR, HER2 or STAT5a/b besides JAK2 whereas Ruxolitinib and BSK-805 are highly selective for JAK proteins [53,54]. Thus, the effects observed upon AG490 treatment could possibly be attributed to the broader spectrum of target molecules of this compound. This observation is very important, since a lower substrate specificity often results in a different response to a drug and is therefore not representative for interfering with a specific molecule which also complicates the identification of predictive biomarker [55]. Also, all bladder cancer cell lines tested by immunoblotting did not show any constitutive phosphorylation of JAK1/2 which might explain the lack of response. In conclusion, our data imply that JAK proteins are not suitable targets for monotherapy in bladder cancer therapy.

Furthermore, we analysed the role of STAT3/5 in bladder cancer cell lines using 3 different STAT3/5 inhibitors Stattic, SH-4-54 and Nifuroxazide. Stattic is a specific STAT3 inhibitor with a lower affinity towards STAT5 [56] and SH-4-54 is a dual inhibitor of STAT3/5 [57]. Nifuroxazide, besides being an inhibitor for STAT3 also inhibits JAK2 and TYK2 [58]. We observed that with these inhibitors proliferation in bladder cancer was greatly reduced which is in line with previous studies on STAT3 inhibition [13,59,60]. Some of the downstream target molecules of STAT3 include anti-apoptotic proteins Bcl-xL. Previously, studies have demonstrated that disrupting STAT3 signalling led to a decrease in anti-apoptotic proteins. Consistent with these findings, we observed a dose-dependent increase in the percentage of apoptotic cells and also a significant increase in cells in G2 phase, which is consistent with prior reports [27].

Combination therapies are a useful approach to overcome limitations of monotherapy options or to enhance the efficiencies. Over the years, there is growing evidence of a connection between STAT3 and chemotherapy resistance [26]. Several studies have shown STAT3 involvement in the development of resistance for chemotherapy in various cancers. It has been demonstrated that STAT3 inhibition sensitized for example squamous cell carcinoma to chemotherapy [61]. In our study, we show that STAT3 inhibition has an additive effect when combined with the most frequently used chemotherapeutic drugs approved for bladder cancer suggesting that this combination might be applicable in patients with STAT3 mediated chemo resistance.

Previously, our group applied a genome wide CRISPR-dCas9 screen to identify acquired resistance mechanisms to the CDK4/6 inhibitor Palbocicib in bladder cancer cell lines [32]. One of the results was an implication of the JAK-STAT signalling pathway in conferring resistance to CDK4/6 inhibitors. The combination therapy with Stattic and Palbociclib showed additive effects in T24 and UMUC-3 but antagonism in RT112 cell lines. Thus, the combination of STAT inhibitors with CDK4/6 inhibitors might not be a general treatment option but should be beneficial for a subset of patients that need to be defined by a detailed analysis of the underlying molecular mechanisms that regulate therapy response in this combination. In conclusion, we show that targeting of STAT3 along with chemotherapy or CDK4/6 inhibitors provide potential combination therapy options in order to improve therapy efficacy in bladder cancer.

In recent years, the development of oncolytic viruses entering clinical trials has dramatically gained momentum [33]. One of the obstacles for inducing an effective therapy response is successful replication of the virus in tumour cells [62]. Thus, a further aim of this study was to combine oncolytic virotherapy with JAK/STAT inhibitors and analyse the effects on virus induced cell death and viral replication. It has been shown that Ruxolitinib enhances replication of VSV but since we could not detect phosphorylation of JAK proteins nor effects on cell viability, we focused on STAT3 inhibitors. Numerous cytokines are released upon virus infection including IFNs and IL-6, which then stimulate the expression of genes that are involved in anti-viral response via direct or indirect mechanisms. It is shown that interferon signalling inhibits adenoviral DNA replication by inhibiting viral early gene expression in normal cells, but not in cancer cells [63,64]. In a STAT2 knockout Syrian hamster model, human adenovirus 5 replicated a 100–1000-fold higher than in the wildtype. The infected cells in the knockout hamsters show interrupted Type I interferon pathway which is implied to be the reason for enhanced replication of virus [65]. Pre-treatment with Ruxolitinib enhanced the viral replication of oncolytic Herpes Simplex Virus (oHSV) in malignant peripheral nerve sheath tumours. Pre-treatment of mice with Ruxolitinib reduced Interferon stimulated genes expression making the tumours susceptible to oHSV infection [42]. Combination of Ruxolitinib and oncolytic vesicular stomatitis virus therapy resulted in enhanced oncolysis and viral replication in non-small cell lung cancer [41].

In this study, combination of STAT3/5 inhibitors with an oncolytic adenovirus resulted in enhancement in virus induced cell death, viral replication and viral particle formation. STAT3/5 inhibition leads to reduction in expression of downstream molecules which also include interleukins and interferons. STAT3/5 inhibition also results in G2 arrest as described. There is evidence for enhanced adenoviral replication upon G2/M arrest [66], which might also be the reason for observed enhancement in viral replication and particle formation upon STAT3/5 inhibition. Further research is required to elucidate the exact mechanisms by which the adenovirus interacts with JAK-STAT pathway and cell cycle. It would also be beneficial to study the viral gene expression analysis to explore the interactions between the viral and cellular genes at various phases of infection.

In conclusion, our study shows that STAT3/5 inhibition reduces cell proliferation both in vitro and in vivo but specific JAK inhibition has no effect on bladder cancer cell lines. We show potential combination therapy options with Stattic and chemotherapeutics, and Stattic and CDK4/6 inhibitor Palbociclib. We also demonstrate enhancement in oncolytic effect of adenoviruses upon combination with STAT3/5 inhibitors. These results indicate that STAT3/5 inhibition, but not JAK1/2 inhibition could be a potentially effective therapeutic strategy in bladder cancer.

## 4. Materials and Methods

### 4.1. Patient Material, Tissue Microarray and Immunohistochemistry

Patient characteristics and the details of tissue microarray and immunohistochemistry have been published before [46].

### 4.2. Cell Lines and Adenovirus

UMUC-3, SW17110, MGHU4, UMUC-6, 639V, SD, J82, VMCUB-1, 647V and BFTC-805 were obtained from Düsseldorf. HCV-29 and KU19-19 were obtained from Leeds, UK. HT1197 was obtained from SIGMA/ECACC, England. BTE-5 were obtained from Uniklinik, Essen. HT-1376, 253J, RT112 and 486P were a kind gift from Homburg. T24, EJ28 and Hek293 cells were from ATCC, VA, USA. Cells were cultured in either RPMI supplemented with 10% foetal bovine serum (FBS), 1% NEAA (Biochrom, Berlin, Germany), penicillin and streptomycin or Dulbecco’s modified Eagle’s medium supplemented with 10% foetal bovine serum and penicillin-streptomycin, at 5% or 10% CO2, respectively. The oncolytic adenovirus XVir-N-31 was kindly provided by Prof. Holm [37].

### 4.3. Small Molecule Inhibitors and Chemotherapeutics

Stock solutions of Ruxolitinib, BSK-805, Stattic, SH-4- 54 and Nifuroxazide (Selleckchem, Munich, Germany) were prepared in dimethyl sulfoxide (DMSO). Palbociclib (Selleckchem, Munich, Germany) stock solution was prepared in water. Chemotherapeutic drugs Paclitaxel, Docetaxel and Gemcitabine (Sigma Aldrich Chemie GmbH, Munich, Germany) stock solutions were prepared in DMSO. Working concentrations were freshly prepared in medium for immediate use. Cisplatin (Sigma Aldrich Chemie GmbH, Munich, Germany) was prepared fresh in H_2_O.

### 4.4. Cell Viability, Cell Cycle Analysis and Apoptosis Assays

Cell viability upon small molecule inhibitors monotherapy, and combination treatment of Stattic and chemotherapy were performed after exposure to inhibitors for indicated time periods by Cell-Titer Blue^®^ assay (Promega, Madison, WI, USA) according to manufacturer’s protocol and absolute IC50 was calculated. For combination treatments of Stattic and Palbociclib and with adenovirus XVir-N-31, a Sulphorhodamine B (SRB) assay was performed. In brief, cells were fixed with 10% trichloroacetic acid, stained with 0.05% SRB and rinsed with 1% acetic acid and allowed to dry. Dried SRB was dissolved in 10 mM tris base and absorbance was measured at 590 nm. These assays were conducted in 12-well plates, seeding 1 × 10^4^ cells/well. For Stattic and Palbociclib treatment, cells were incubated for 3 days post treatment.

The effect of virus induced cell killing in combination with small molecule inhibitors was analysed in 12-well plates. In total, 0.25–0.5 × 10^5^ cells were seeded and infected with increasing concentrations (multiplicity of infection, MOI) of the adenovirus XVir-N-31 one day after treatment with Stattic in 200µl medium without FBS. At 1hpi, complete medium with or without Stattic was added to the cells. Cells were fixed 4 days post infection and cell viability was analysed by SRB assay.

Apoptosis (Caspase-Glo^®^ 3/7 Assay-Promega) and cell cycle analysis (7-AAD, Thermo Fisher Scientific) were performed according to the manufacturer’s protocol.

### 4.5. Combination Index Analysis and Bladder Cancer Molecular Alteration Analysis

For combination therapy with Stattic and chemotherapeutics, fixed ratio combinations were used. The fixed ratio combinations are as follows: For T24 cells: S:P (Stattic: Paclitaxel) at 122.2:1, S:C (Stattic and Cisplatin) at 1:2.1, S:G (Stattic and Gemcitabine) at 1:2.1 and S:D (Stattic: Docetaxel) at 1447.4:1; for J82 cells: S:P (Stattic: Paclitaxel) at 33.3:1, S:C (Stattic and Cisplatin) at 1:1, S:G (Stattic and Gemcitabin) at 25:1 and S:D (Stattic: Docetaxel) at 250:1; for HT1376 cells: S:P(Stattic:Paclitaxel) at 176:1, S:C (Stattic and Cisplatin) at 1:5.68, S:G (Stattic and Gemcitabine) at 149.15:1 and S:D (Stattic: Docetaxel) at 352:1; for RT112 cells: S:P(Stattic:Paclitaxel) at 500:1, S:C (Stattic and Cisplatin) at 1:1, S:G (Stattic and Gemcitabine) at 50:1 and S:D (Stattic: Docetaxel) at 2000:1; for SD cells: S:P(Stattic:Paclitaxel) at 444.4:1, S:C (Stattic and Cisplatin) at 1:1, S:G (Stattic and Gemcitabine) at 160:1 and S:D (Stattic: Docetaxel) at 4000:1. The combination index (CI) was assessed with the Chou–Talalay combination index (CI) theorem [51]. CI value of 1 was defined as additivity, CI < 1 as synergistic and CI >1 as antagonistic effects. The analysis was performed with CompuSyn software (Combosyn, NJ, USA). The Cancer Genome Atlas (TCGA) analysis was performed on cBioPortal (https://www.cbioportal.org/).

### 4.6. Immunoblot Analysis

Cells were seeded in 10 cm tissue culture plates and allowed to grow till 50–60% confluency. The medium was then changed with FBS-free medium overnight. Stattic was applied in indicated concentrations for 3 h and cells were stimulated by supplementing with IL-6 (25ng/mL) for 30 min. Then protein extraction (either total or compartmental) was performed. Cells were lysed on ice in protein lysis buffer comprising of 1% sodium dodecyl sulphate, 1 mM sodium orthovanadate and 10 mM tris, pH 7.4. Lysates were sheared using a 27-gauge needle (BD Biosciences^®^) until no viscosity was observed. The sheared lysates were then centrifuged at 30,000 RCF at 4 °C for total lysates. For compartmentalisation, cells were harvested by trypsin, pelleted then washed twice in phosphate-buffered saline, pelleted again by spinning at 600 RCF in microcentrifuge at 4 °C for 4 min. The pellet was resuspended in lysis buffer. After 5 min on ice, the lysates were spun at 600 RCF in microcentrifuge at 4 °C for 4 min. The supernatants were used as cytoplasmic extracts. The pelleted nuclei were briefly washed in lysis buffer without NP-40. The nuclear pellet was then resuspended in 50–100 μL nuclear extract buffer (20 mM Tris-HCl (pH 8.0), 420 mM NaCl, 1.5 mM MgCl_2_, 0.2 mM EDTA, 25% glycerol) After a 10-min incubation at 4 °C, the nuclei were briefly vortexed and spun at 18300 RCF in microcentrifuge at 4 °C for 15 min. The supernatant was then removed and used as a nuclear extract. The primary antibodies used included: Stat3 (#9139), p-Stat3 (#9145), Jak1 (#3344), p-Jak1 (#3331), Jak2 (#3230), p-Jak2 (#3771), cyclin-D1(#2922) and GAPDH (#2118) from Cell Signaling Technology, Danvers, MA, USA. Actin (#A2066) from Sigma, Saint Louis, Missouri, USA and histone H1 (#05-457) from Millipore corporation, Temecula, CA, USA.

### 4.7. CAM Assay and Immunohistochemistry (IHC)

The CAM assay was performed as described previously [50]. In brief, 2 million cells were seeded on embryonic day 8 on the CAM. Topical treatment using 2 µM Stattic was performed daily from day 10 after the formation of visible xenografts. The control tumours were treated with PBS. On day 15, the tumours were harvested: embryos were transferred to a Styrofoam box containing dry ice and suffocated. Tumours were then removed from the CAM, immersed in a petri dish filled with ice-cold PBS and trimmed under a stereo microscope (Leica) with micro scissors to remove as much of the attached CAM as possible. The tumours were immersed in pre-weighed 1.5 mL reaction tubes containing PBS and weighed on a fine balance. IHC was done with Ki-67 (No. M7240, Dako Deutschland, Hamburg, Germany) using heat induced epitope retrieval with 0.01 M citrate buffer at pH 6.0.

### 4.8. Viral Replication and Particle Formation

Thus, 5 × 10^4^ cells were seeded in 6-well plates and treated with specified concentrations of Stattic. Viral infection was performed a day after the inhibitor treatment in the same manner as described for cell viability assay. DNA lysates were made 4–48 h after infection for viral replication analysis and lysates and supernatant were collected at 72 h for viral particle formation analysis. Viral replication was analysed by real time PCR for fibre DNA using the ΔΔCT-method. Actin was used as the housekeeping gene. Viral titres in the lysates were measured in Hek293 cells seeded in 24-well plates by hexon staining. The protocols for viral replication analysis and hexon titre test were performed as described [37].

### 4.9. Statistical Analysis

All the cell error bars in cell viability assays, apoptosis assays are presented as mean ± standard error. Statistical analysis for patient’s survival analysis and CAM assay was performed using SPSS software. *p* < 0.05 was considered as significant.

## Supplementary Materials

Supplementary materials can be found at https://www.mdpi.com/1422-0067/21/3/1106/s1. Dose response curves for individual cell lines for Ruxolitinib (Figure S1), BSK-805 (Figure S2), SH-4-54 (Figure S3), Nifuroxazide (Figure S4) and Stattic (Figure S5) are generated 72 h after treatment. Dose response curves for the combination therapy of Stattic and chemotherapeutics are generated 72h after treatment (Figure S6).Molecular alterations in the JAK-STAT signalling pathway related genes in bladder cancer in TCGA (Table S1) and co-occurrence of altered genes (Table S2) are analysed using cBioPortal.

## Abbreviations

CAM	Chorioallantoic Membrane
FGFR	Fibroblast Growth Factor Receptor
JAK	Janus Kinase
STAT	Signal Transducer and Activator of Transcription
TCGA	The Cancer Genome Atlas
