# Supplementary material for: STAT3/5 Inhibitors Suppress Proliferation in Bladder Cancer and Enhance Oncolytic Adenovirus Therapy
Source: Int J Mol Sci. 2020 Feb 7;21(3):1106. doi: 10.3390/ijms21031106 (PMC7043223; doi:10.3390/ijms21031106)
Supplement: Supplementary file 1 [file ijms-21-01106-s001.zip › Supplementary-reviewed-PDF/JAK STAT in Bladder cancer-Supplementary information_review.pdf]

**Supplementary figure-S1:** JAK inhibition by specific inhibitors: Cells were treated with increasing concentrations of Ruxolitinib and cell viability was assessed by CellTiter-Blue® Cell Viability Assay 72 hours after treatment in a panel of bladder cancer cell lines: Error bars S.E.

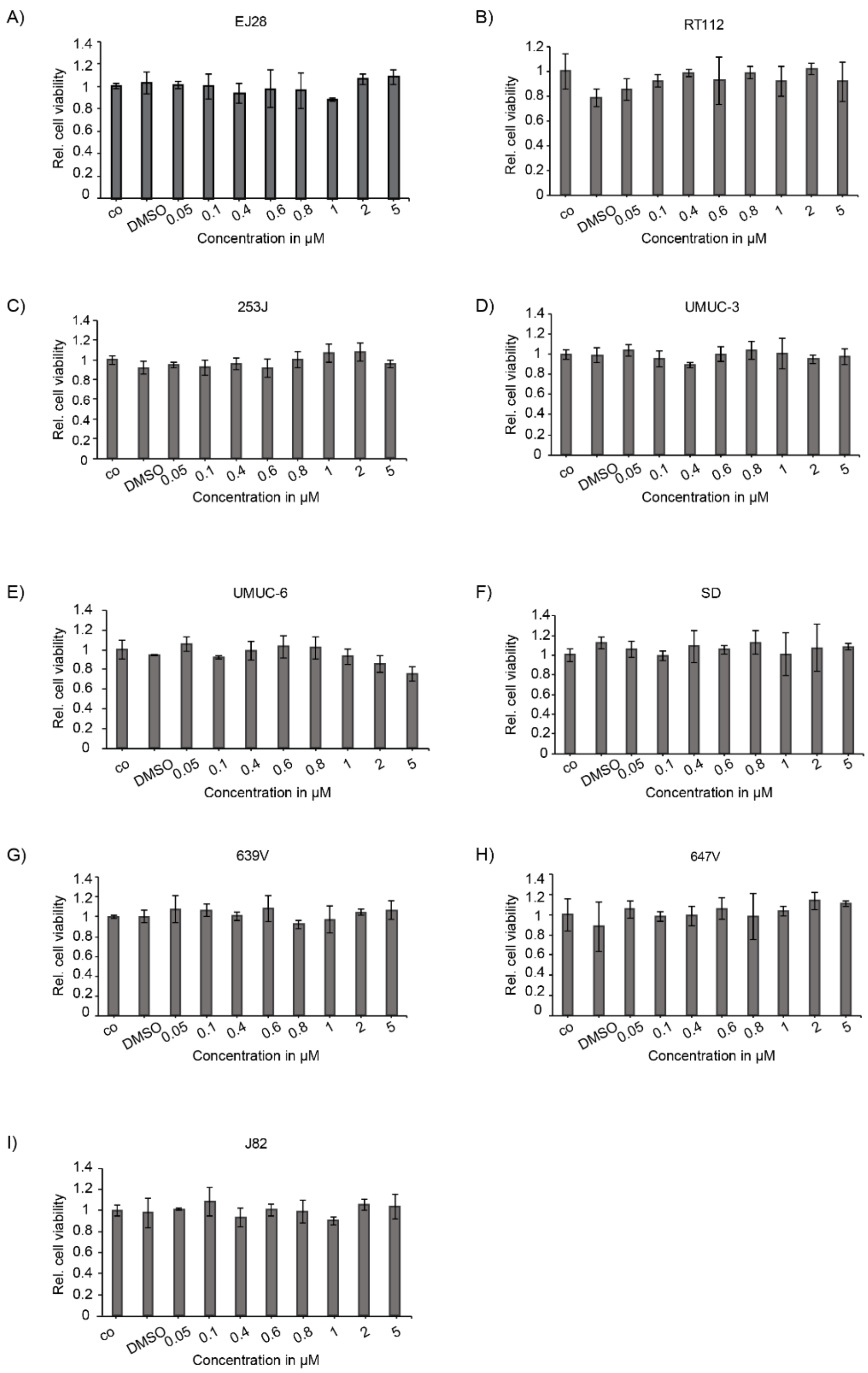

**Supplementary figure-S2:** JAK inhibition by specific inhibitors: Cells were treated with increasing concentrations of BSK-805 and cell viability was assessed by CellTiter-Blue® Cell Viability Assay 72 hours after treatment in a panel of bladder cancer cell lines: Error bars S.E.

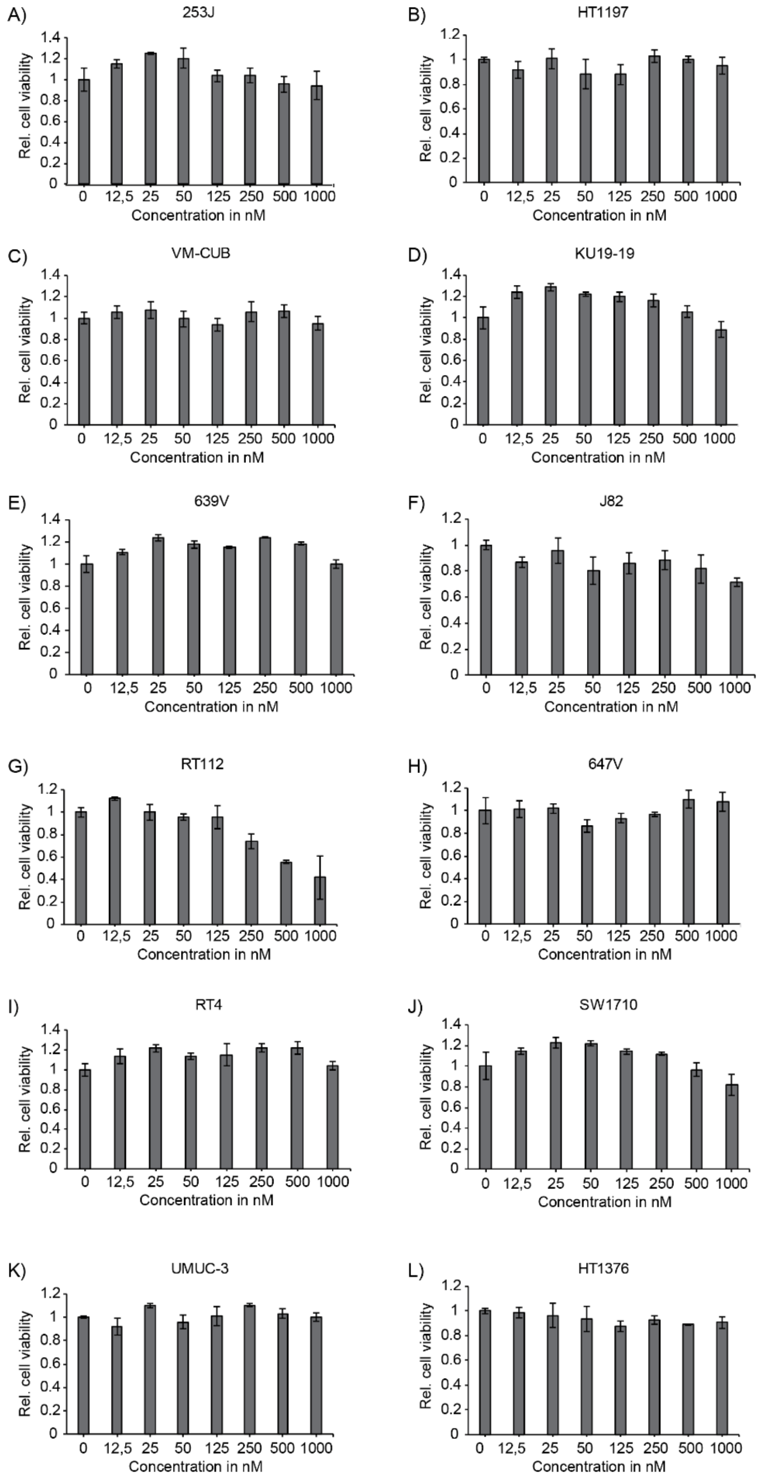

**Supplementary figure-S3:** STAT3/5 inhibition by specific inhibitors: Cells were treated with increasing concentrations of SH-4-54 and cell viability was assessed by CellTiter-Blue® Cell Viability Assay 72 hours after treatment in a panel of bladder cancer cell lines: Error bars S.E.

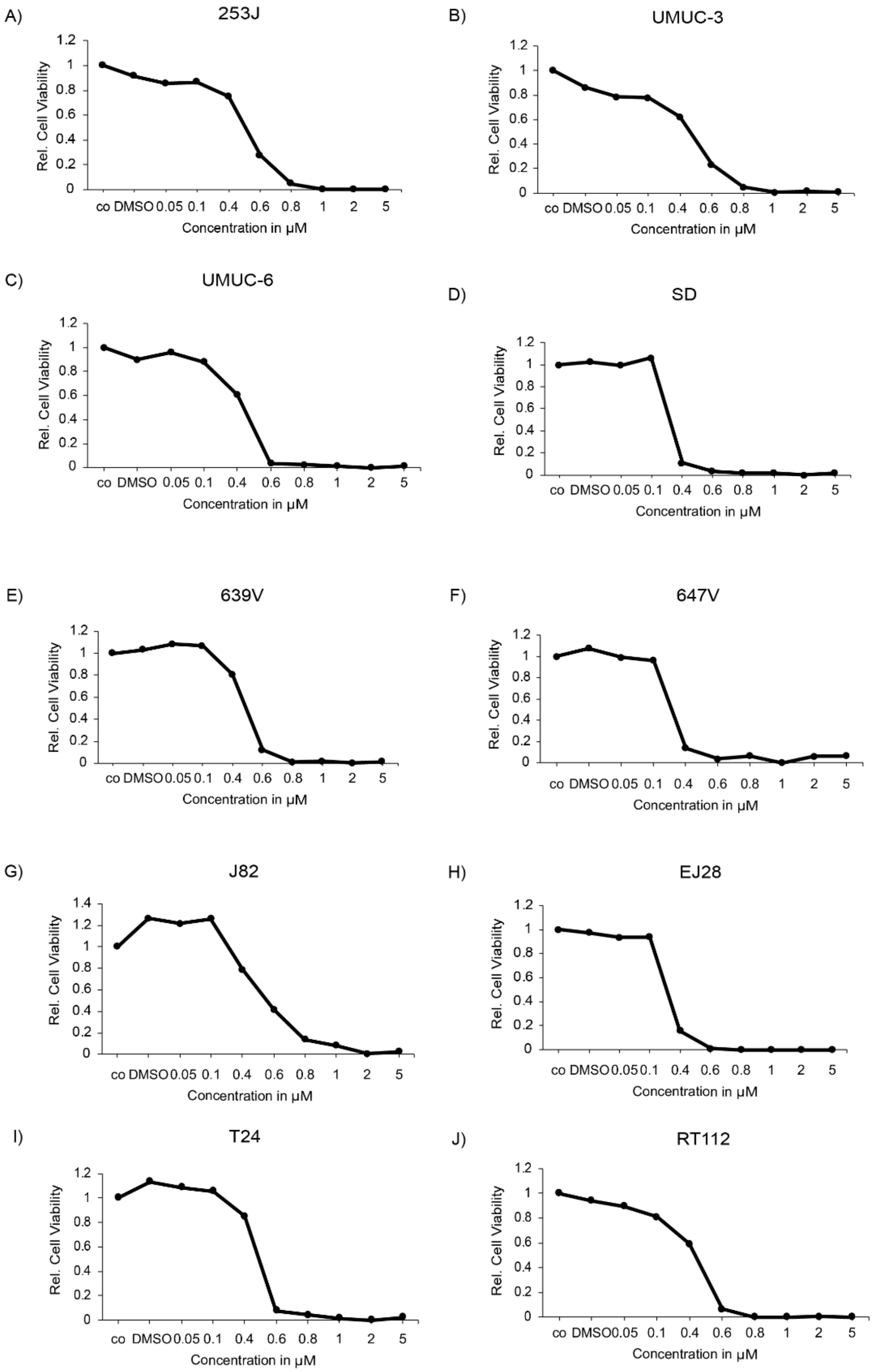

**Supplementary figure-S4:** STAT3/5 inhibition by specific inhibitors: Cells were treated with increasing concentrations of Nifuroxazide and cell viability was assessed by CellTiter-Blue® Cell Viability Assay 72 hours after treatment inT24 (A) and RT112 (B) cells. Error bars S.E.

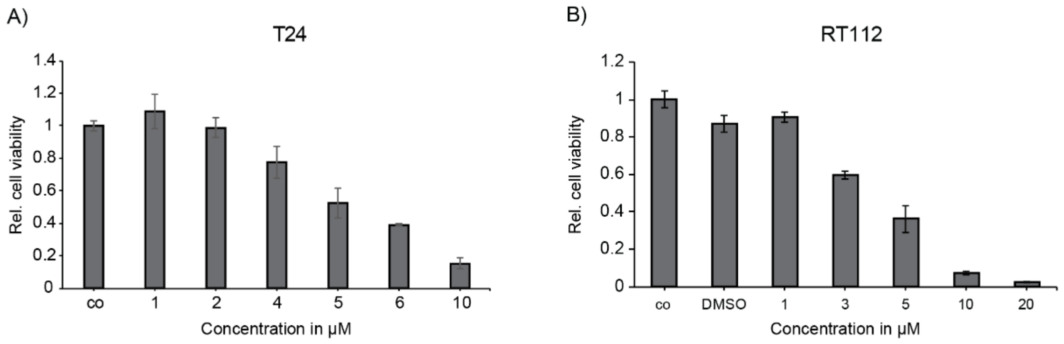

**Supplementary figure-S5:** STAT3/5 inhibition by specific inhibitors: Cells were treated with increasing concentrations of Stattic and cell viability was assessed by CellTiter-Blue® Cell Viability Assay 72 hours after treatment in a panel of bladder cancer cell lines: Error bars S.E.

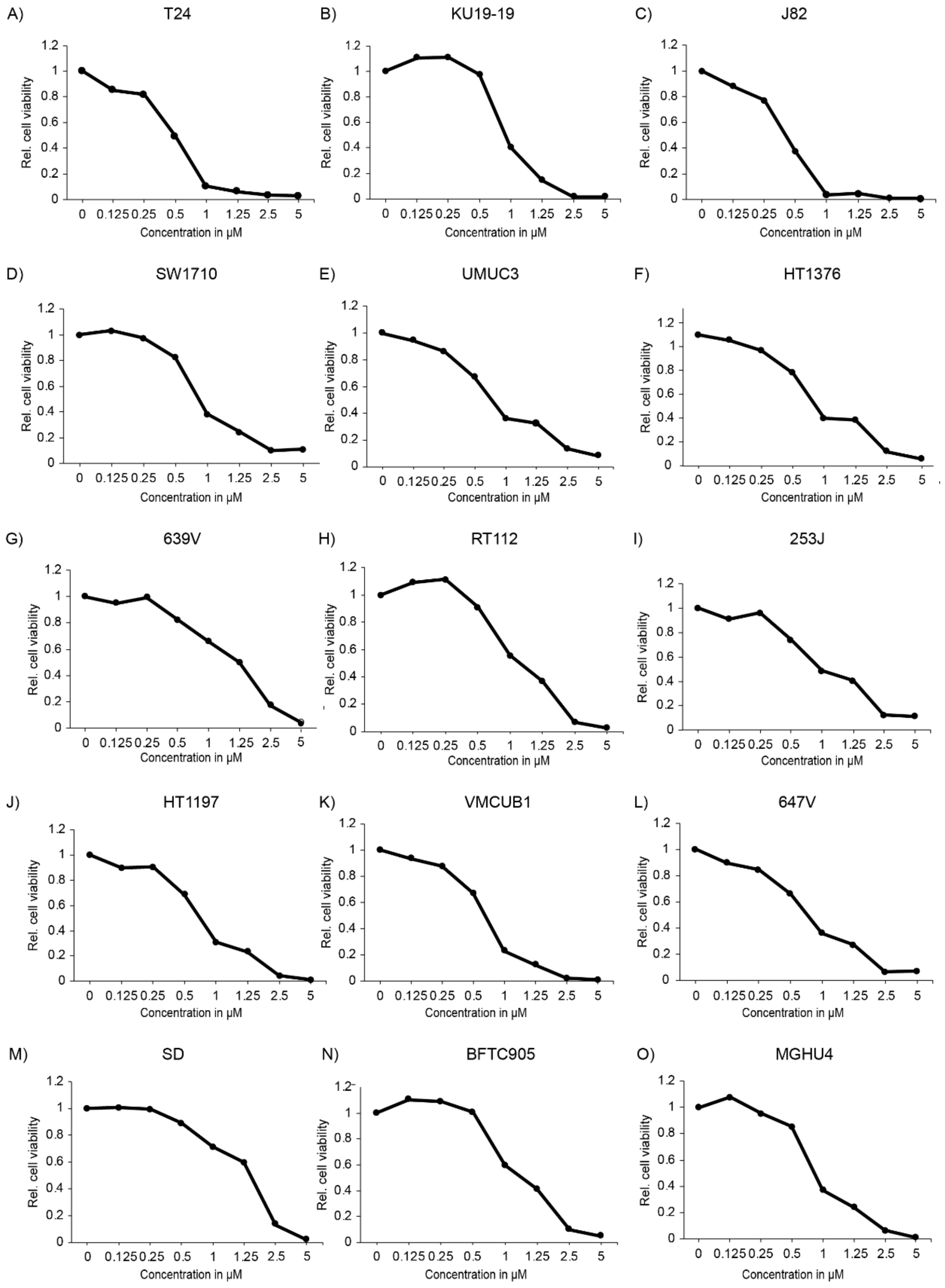

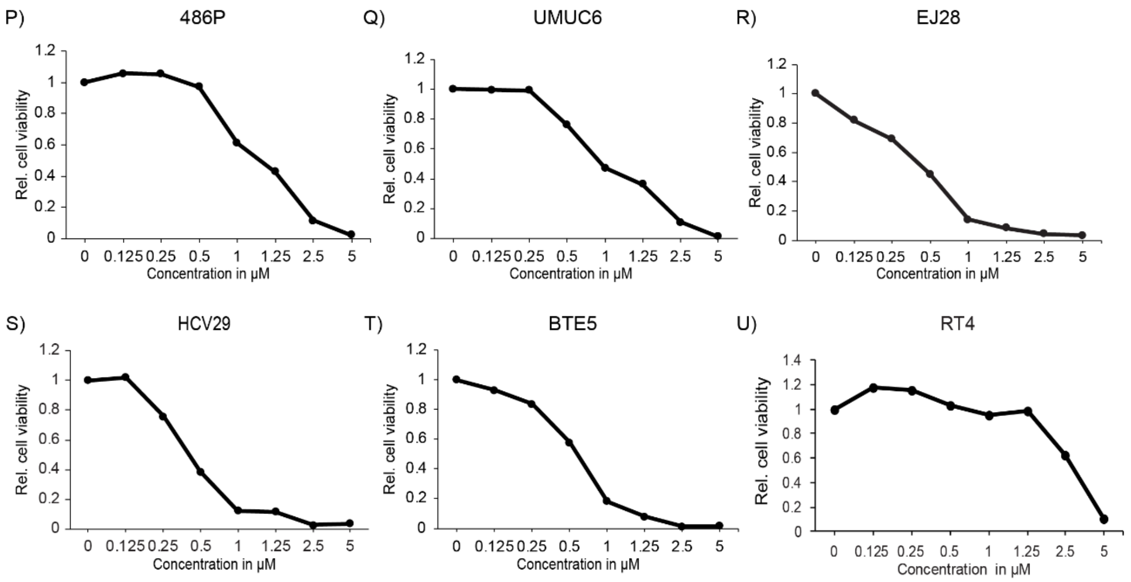
P)
486P
Rel. cell viability
Concentration in µM
Q)
UMUC6
Rel. cell viability
Concentration in µM
R)
EJ28
Rel. cell viability
Concentration in µM
S)
HCV29
Rel. cell viability
Concentration in µM
T)
BTE5
Rel. cell viability
Concentration in µM
U)
RT4
Rel. cell viability
Concentration in µM
0 0.125 0.25 0.5 1 1.25 2.5 5

**Supplementary figure-S6:** Dose response curves for combination therapy with Stattic and chemotherapeutics: Treatment of the respective cell lines was done for 72 hours with Stattic alone and in a fixed ratio combination with Paclitaxel, Cisplatin, Gemcitabine or Docetaxel in T24, J82, HT1376, RT112, and SD cell lines (See Materials and methods) and and cell viability was assessed by CellTiter-Blue® Cell Viability Assay 72 hours after treatment. Error bars S.E.

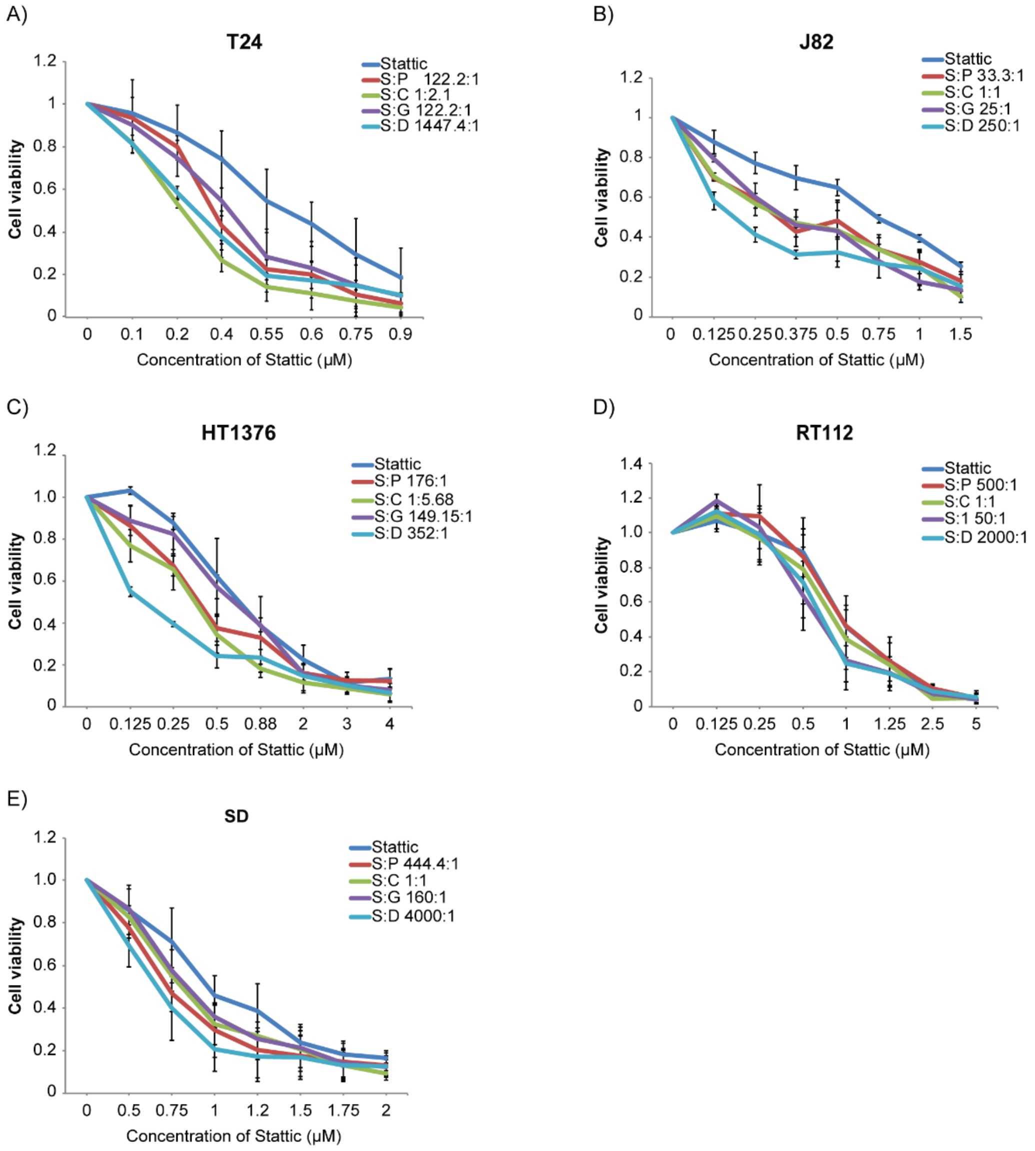

**Supplementary table-1:** Alterations in the JAK-STAT signalling pathway related genes in bladder cancer in the TCGA dataset.

| S.No | Gene symbol | Alteration in gene expression (n= 412) | |
|---|---|---|---|
| | | Number of cases with alterations | % |
| 1 | JAK1 | 29 | 7 |
| 2 | JAK2 | 54 | 13 |
| 3 | JAK3 | 32 | 8 |
| 4 | TYK2 | 28 | 7 |
| 5 | STAT1 | 42 | 10 |
| 6 | STAT2 | 21 | 5 |
| 7 | STAT3 | 29 | 7 |
| 8 | STAT4 | 33 | 8 |
| 9 | STAT5a | 38 | 9 |
| 10 | STAT5b | 24 | 6 |
| 11 | STAT6 | 27 | 7 |

**Supplementary table-2:** Co-occurrence of altered genes in the JAK-STAT pathway in the TCGA dataset.

| Gene A | Gene B | Neither A nor B | A Not B | B Not A | Both A and B | Log2 Odds Ratio | p-Value | Tendency |
|---|---|---|---|---|---|---|---|---|
| STAT3 | STAT5A | 289 | 15 | 22 | 10 | >3 | <0.001 | Co-occurrence |
| JAK2 | STAT5A | 263 | 41 | 18 | 14 | 2.319 | <0.001 | Co-occurrence |
| STAT3 | STAT5B | 294 | 18 | 17 | 7 | 2.75 | <0.001 | Co-occurrence |
| STAT5A | STAT5B | 287 | 25 | 17 | 7 | 2.241 | 0.004 | Co-occurrence |
| JAK2 | STAT3 | 265 | 46 | 16 | 9 | 1.696 | 0.011 | Co-occurrence |
| JAK1 | STAT3 | 289 | 22 | 19 | 6 | 2.053 | 0.011 | Co-occurrence |
