# Supplementary material for: STAT3/5 Inhibitors Suppress Proliferation in Bladder Cancer and Enhance Oncolytic Adenovirus Therapy
Source: Int J Mol Sci. 2020 Feb 7;21(3):1106. doi: 10.3390/ijms21031106 (PMC7043223; doi:10.3390/ijms21031106)
Supplement: Supplementary file 1 [file ijms-21-01106-s001.zip › Supplementary-reviewed-PDF/Supplementary table-1.pdf]

S.No	Gene symbol	Alteration in gene expression (n= 412)	
		Number of cases with alterations	%
1	JAK1	29	7
2	JAK2	54	13
3	JAK3	32	8
4	TYK2	28	7
5	STAT1	42	10
6	STAT2	21	5
7	STAT3	29	7
8	STAT4	33	8
9	STAT5a	38	9
10	STAT5b	24	6
11	STAT6	27	7
