# Supplementary material for: STAT3/5 Inhibitors Suppress Proliferation in Bladder Cancer and Enhance Oncolytic Adenovirus Therapy
Source: Int J Mol Sci. 2020 Feb 7;21(3):1106. doi: 10.3390/ijms21031106 (PMC7043223; doi:10.3390/ijms21031106)
Supplement: Supplementary file 1 [file ijms-21-01106-s001.zip › Supplementary-reviewed-PDF/Supplementary table-2.pdf]

Gene A	Gene B	Neither A nor B	A Not B	B Not A	Both A and B	Log2 Odds Ratio	p-Value	Tendency
STAT3	STAT5A	289	15	22	10	>3	<0.001	Co-occurrence
JAK2	STAT5A	263	41	18	14	2.319	<0.001	Co-occurrence
STAT3	STAT5B	294	18	17	7	2.75	<0.001	Co-occurrence
STAT5A	STAT5B	287	25	17	7	2.241	0.004	Co-occurrence
JAK2	STAT3	265	46	16	9	1.696	0.011	Co-occurrence
JAK1	STAT3	289	22	19	6	2.053	0.011	Co-occurrence
