# Supplementary figures and images for: STAT3/5 Inhibitors Suppress Proliferation in Bladder Cancer and Enhance Oncolytic Adenovirus Therapy
Source: Int J Mol Sci. 2020 Feb 7;21(3):1106. doi: 10.3390/ijms21031106 (PMC7043223; doi:10.3390/ijms21031106)

A)

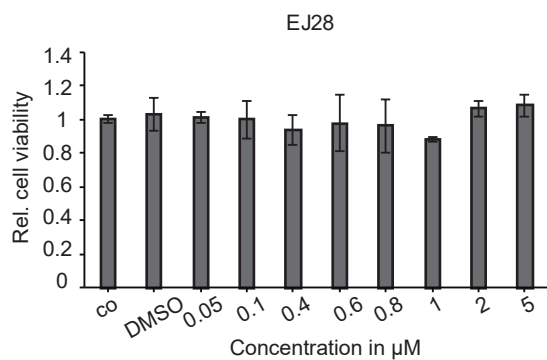

B)

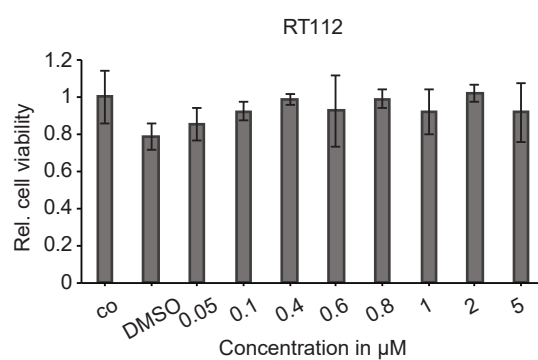

C)

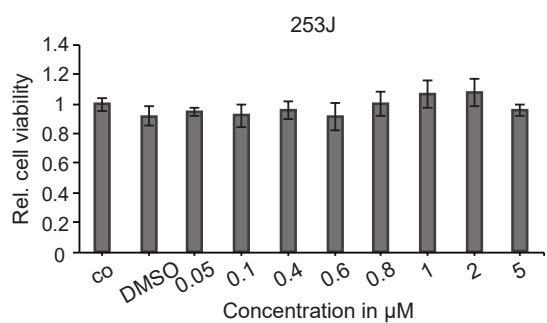

D)

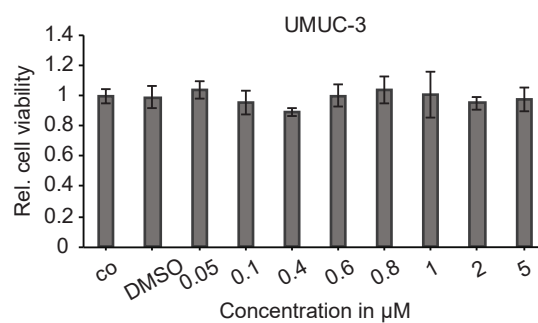

E)

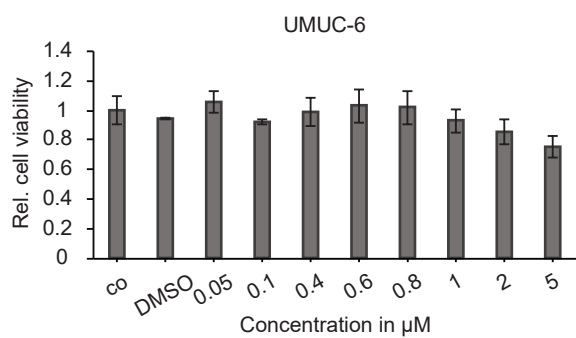

F)

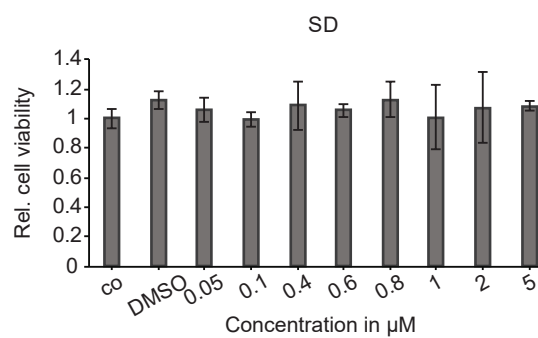

G)

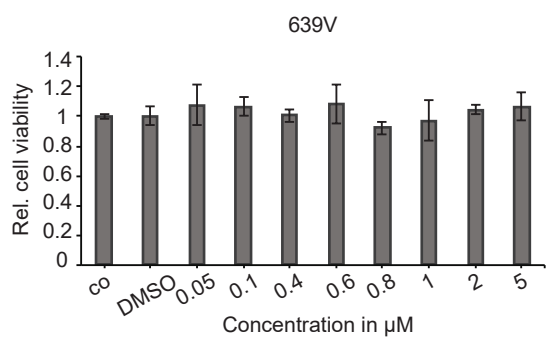

H)

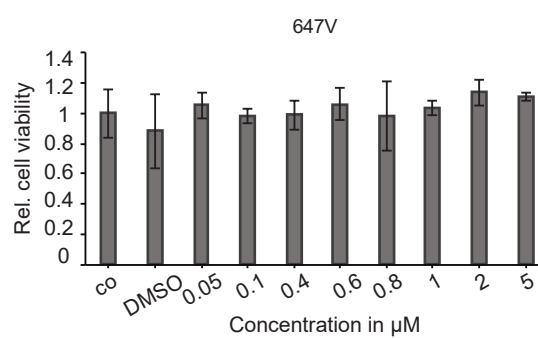

I)

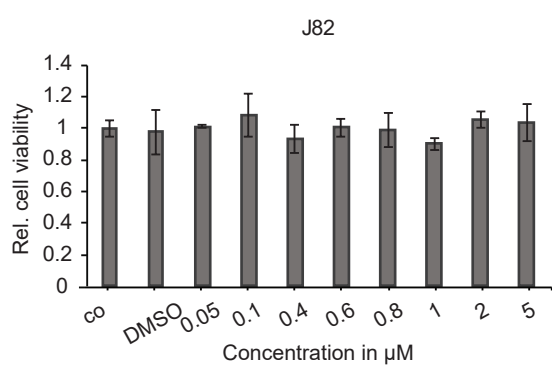

Supplement: Supplementary file 1 [file ijms-21-01106-s001.zip › Supplementary-reviewed-PDF/Figure-S1.pdf]

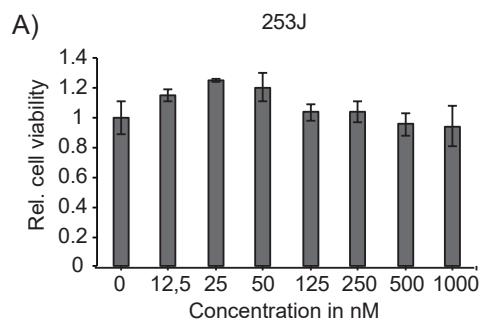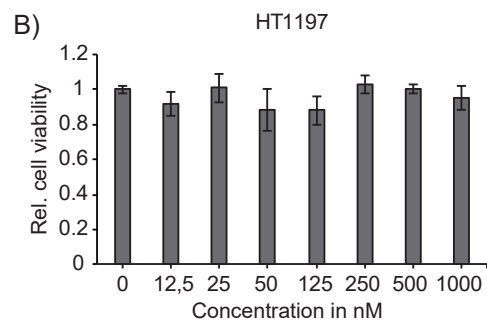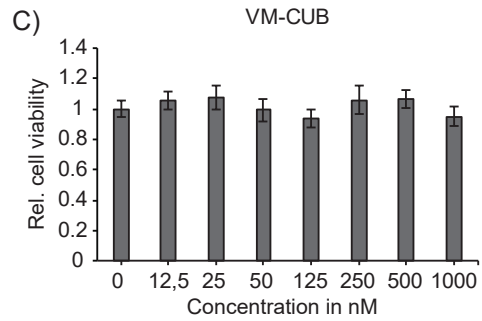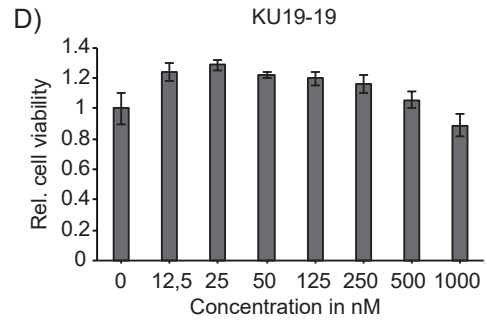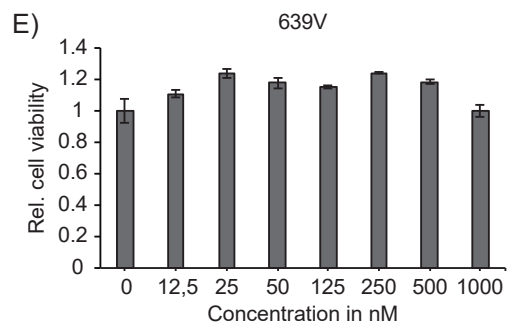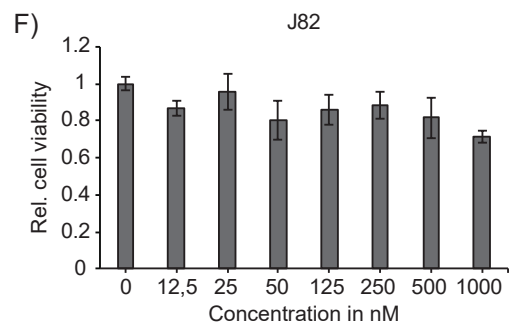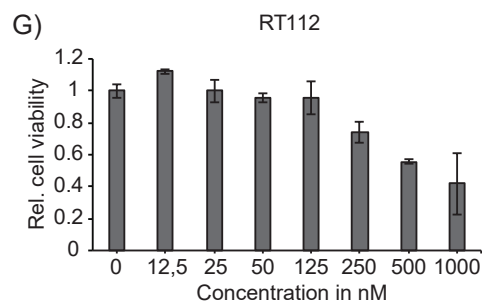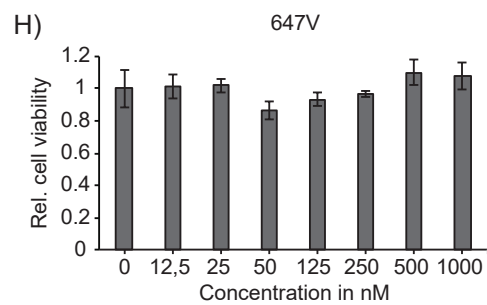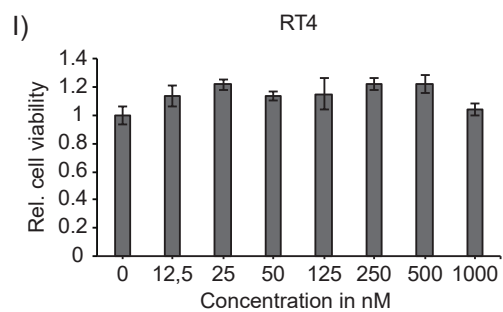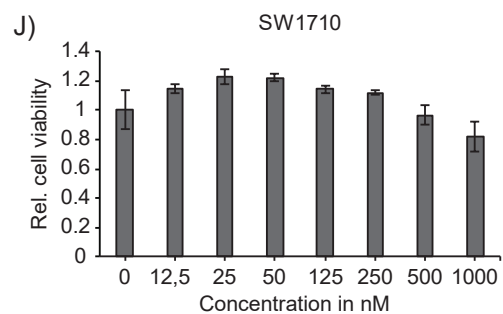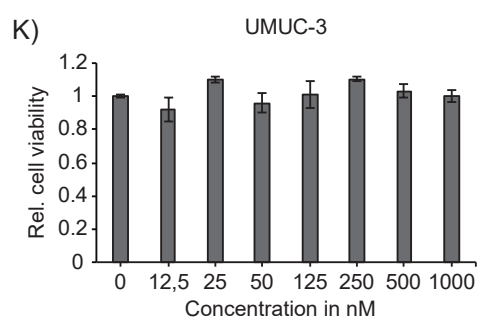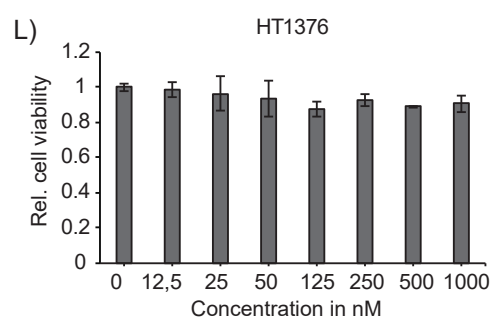

Supplement: Supplementary file 1 [file ijms-21-01106-s001.zip › Supplementary-reviewed-PDF/Figure-S2.pdf]

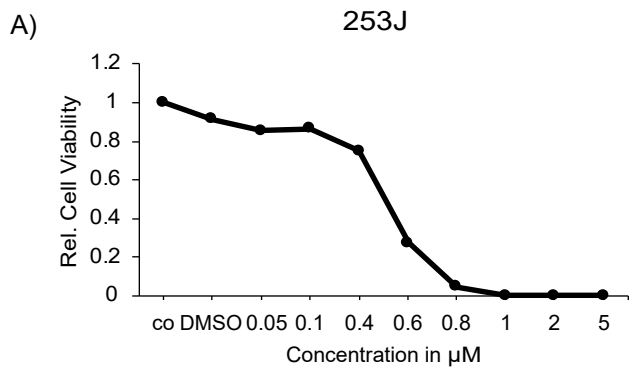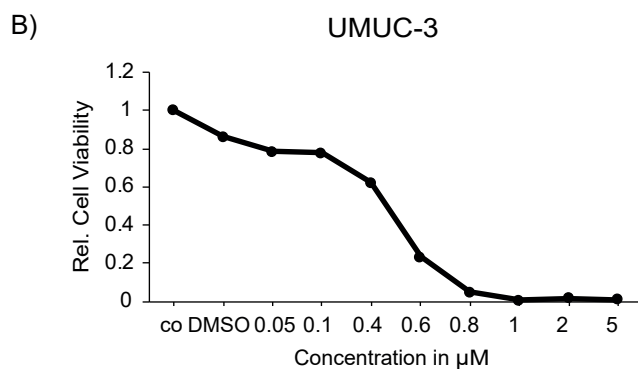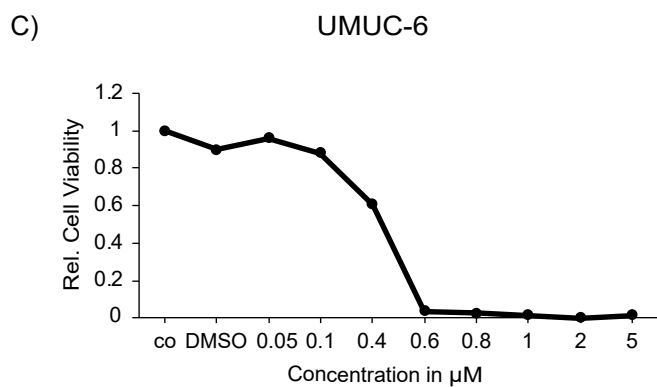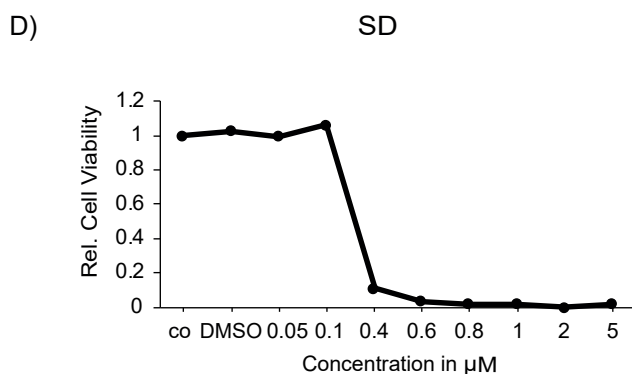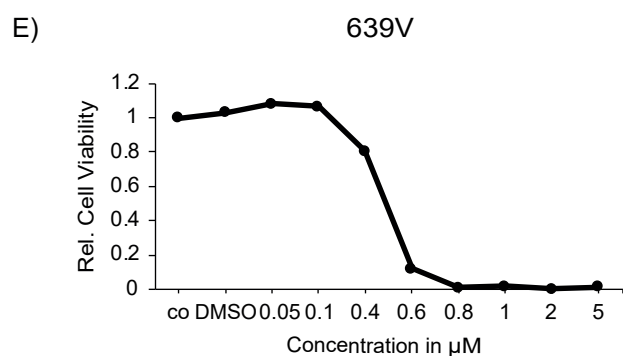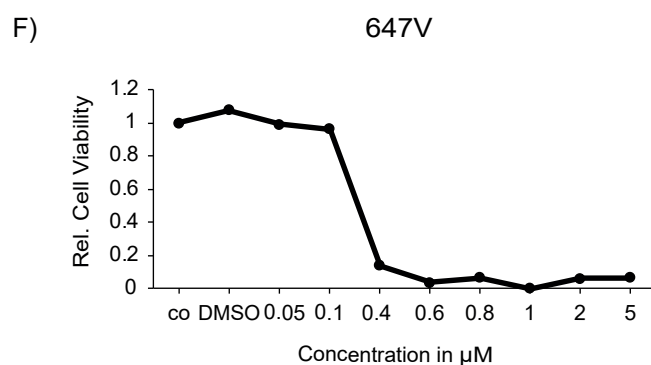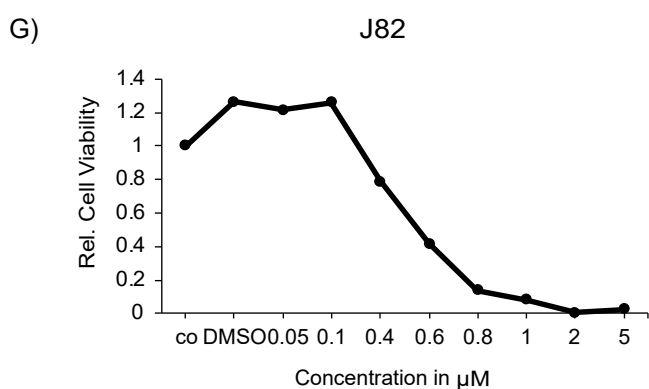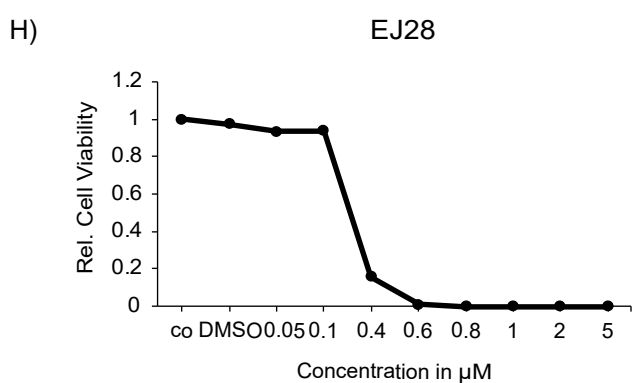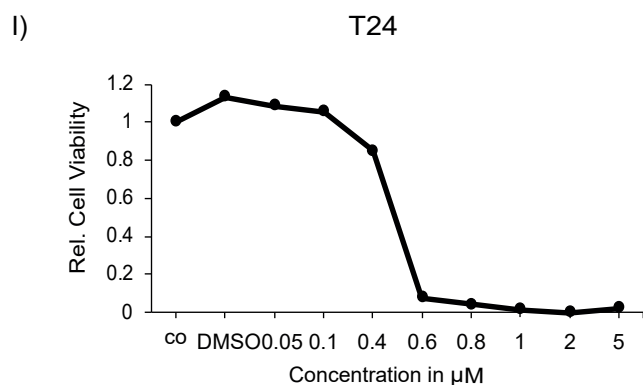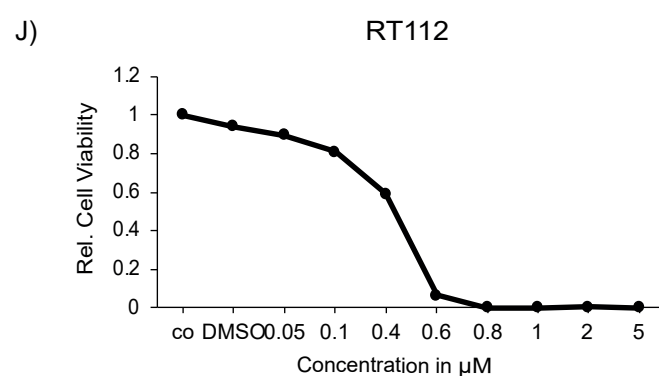

Supplement: Supplementary file 1 [file ijms-21-01106-s001.zip › Supplementary-reviewed-PDF/Figure-S3.pdf]

A)

T24

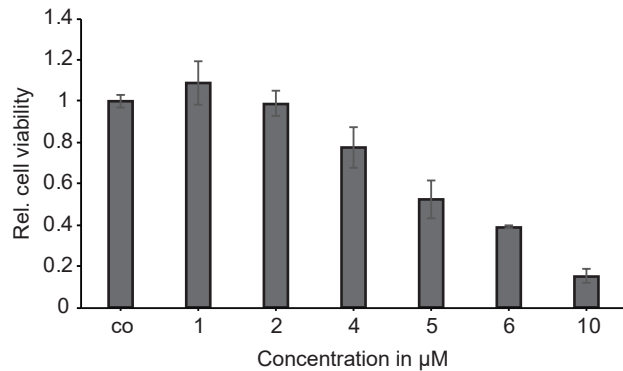

B)

RT112

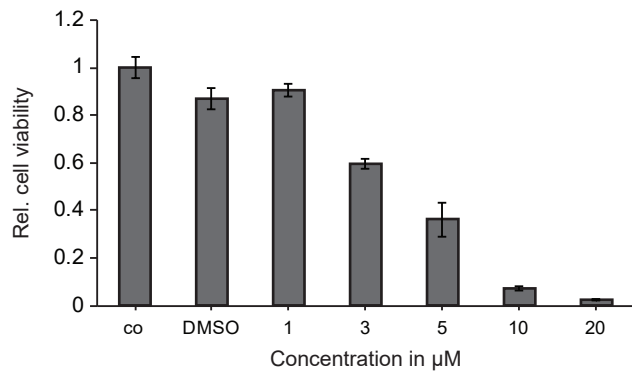

Supplement: Supplementary file 1 [file ijms-21-01106-s001.zip › Supplementary-reviewed-PDF/Figure-S4.pdf]

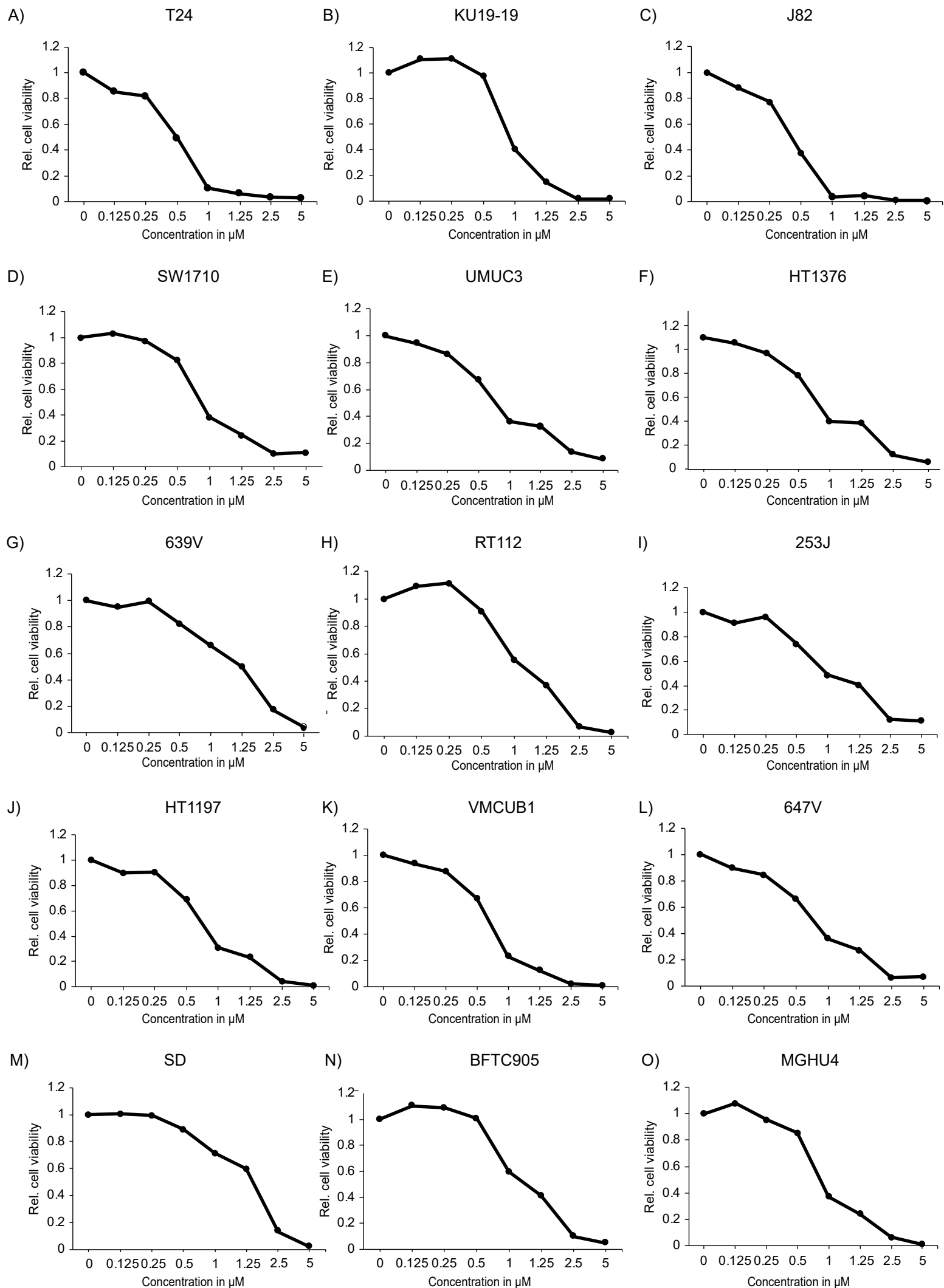

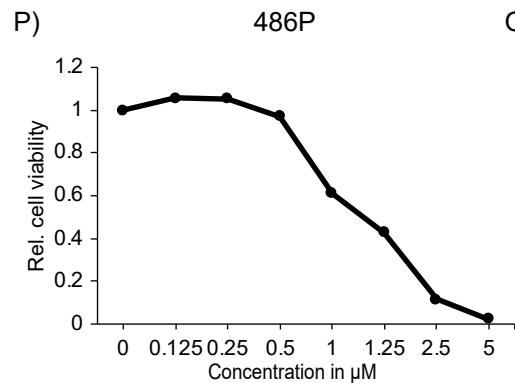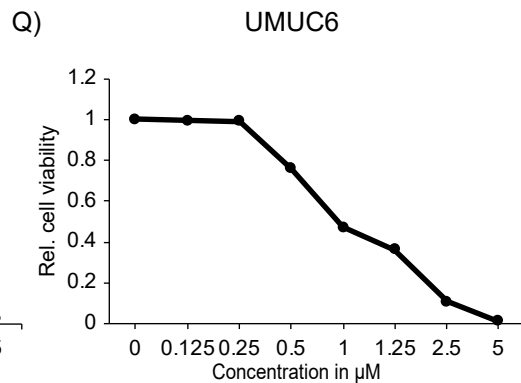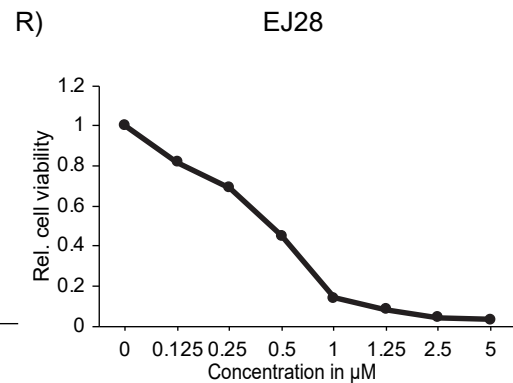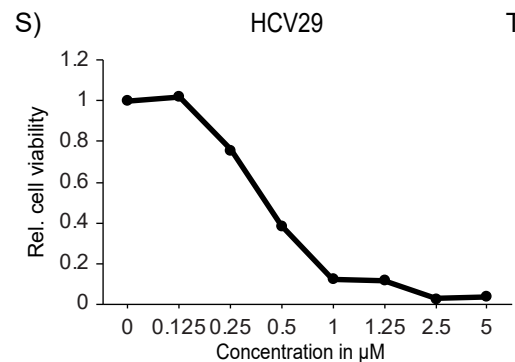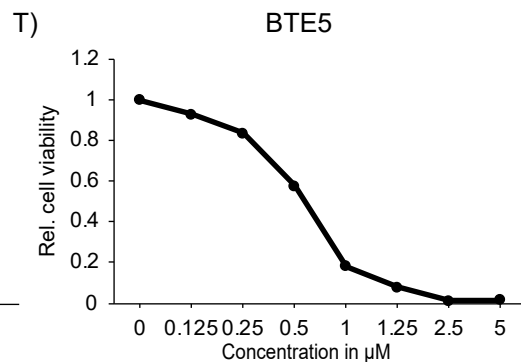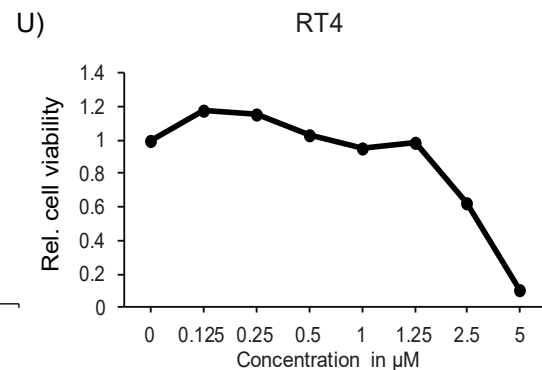

Supplement: Supplementary file 1 [file ijms-21-01106-s001.zip › Supplementary-reviewed-PDF/Figure-S5.pdf]

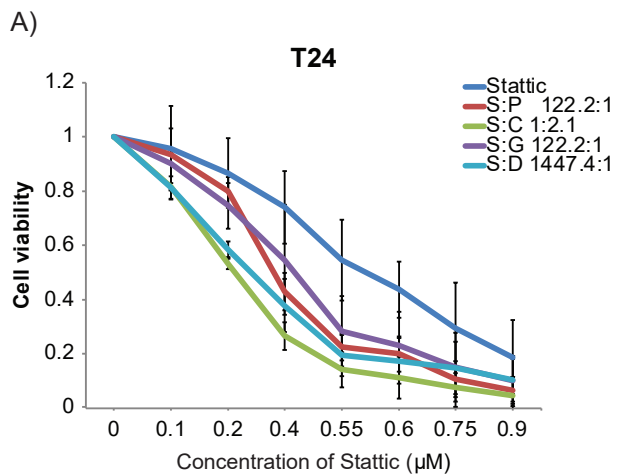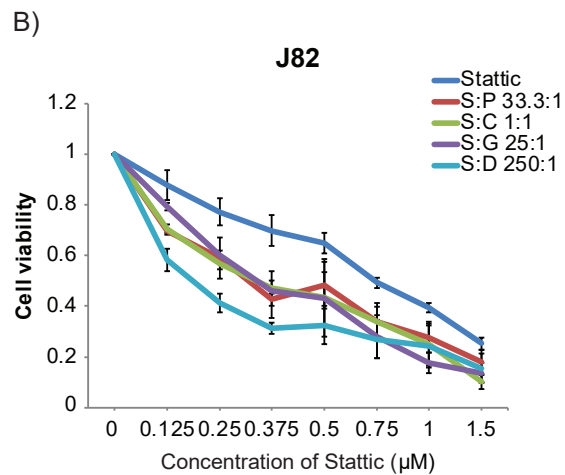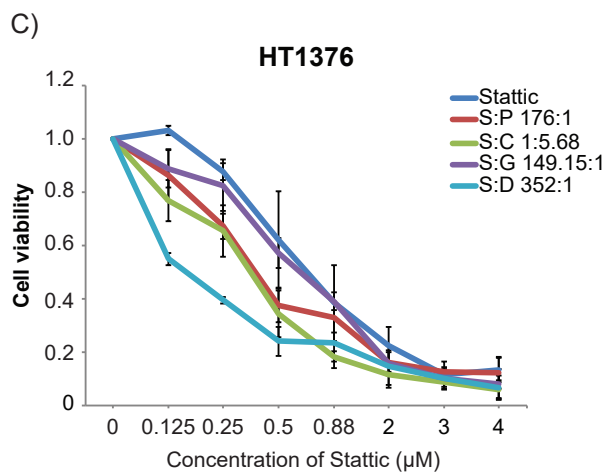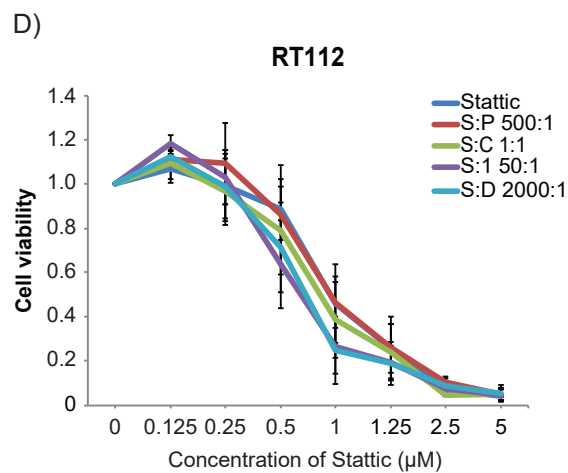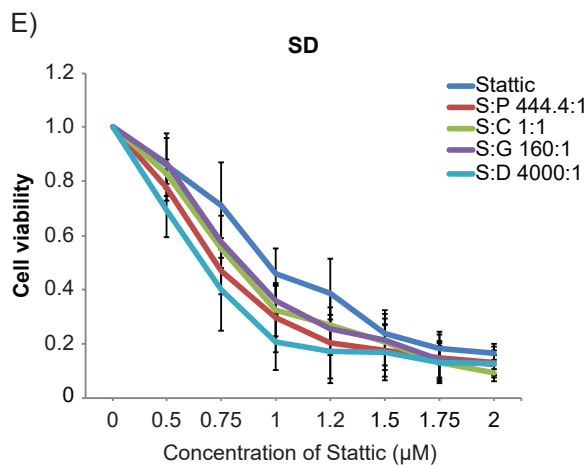

Supplement: Supplementary file 1 [file ijms-21-01106-s001.zip › Supplementary-reviewed-PDF/Figure-S6.pdf]
